# Evaluation of therapeutic effects of tetramethylpyrazine nitrone in Alzheimer’s disease mouse model and proteomics analysis

**DOI:** 10.3389/fphar.2023.1082602

**Published:** 2023-03-06

**Authors:** Xinhua Zhou, Kaipeng Huang, Yuqiang Wang, Zaijun Zhang, Yingying Liu, Qinghua Hou, Xifei Yang, Maggie Pui Man Hoi

**Affiliations:** ^1^ Department of Neurology and Stroke Center, Jinan University College of Pharmacy, The First Affiliated Hospital of Jinan University and Institute of New Drug Research, Guangzhou, China; ^2^ State Key Laboratory of Quality Research in Chinese Medicine, Institute of Chinse Medical Sciences, University of Macau, Macau, China; ^3^ Institute of GCP, Guangzhou Eighth People’s Hospital Guangzhou Medical University, Guangzhou, China; ^4^ Guangdong Province Key Laboratory of Pharmacodynamic, College of Pharmacy, Institute of New Drug Research, Constituents of Traditional Chinese Medicine & New Drug Research, Jinan University, Guangdong, China; ^5^ Department of Neurology, Daqing People’s Hospital, Daqing, China; ^6^ Department of Neurology, Clinical Neuroscience Center, the 7th Affiliated Hospital, Sun-Yat-sen University. Shenzhen, China; ^7^ Key Laboratory of Modern Toxicology of Shenzhen, Shenzhen Center for Disease Control and Prevention, Shenzhen, China; ^8^ DPS, Faculty of Health Sciences, University of Macau, Macau, China

**Keywords:** alhzheimer disease, tetramethylpyrazine nitrone, amyloid beta, proteomic analysis, PINK1

## Abstract

The pathophysiology of Alzheimer’s disease (AD) is multifactorial with characteristic extracellular accumulation of amyloid-beta (Aβ) and intraneuronal aggregation of hyperphosphorylated tau in the brain. Development of disease-modifying treatment for AD has been challenging. Recent studies suggest that deleterious alterations in neurovascular cells happens in parallel with Aβ accumulation, inducing tau pathology and necroptosis. Therefore, therapies targeting cellular Aβ and tau pathologies may provide a more effective strategy of disease intervention. Tetramethylpyrazine nitrone (TBN) is a nitrone derivative of tetramethylpyrazine, an active ingredient from *Ligusticum wallichii Franchat* (Chuanxiong). We previously showed that TBN is a potent scavenger of free radicals with multi-targeted neuroprotective effects in rat and monkey models of ischemic stroke. The present study aimed to investigate the anti-AD properties of TBN. We employed AD-related cellular model (N2a/APPswe) and transgenic mouse model (3×Tg-AD mouse) for mechanistic and behavioral studies. Our results showed that TBN markedly improved cognitive functions and reduced Aβ and hyperphosphorylated tau levels in mouse model. Further investigation of the underlying mechanisms revealed that TBN promoted non-amyloidogenic processing pathway of amyloid precursor protein (APP) in N2a/APPswe *in vitro*. Moreover, TBN preserved synapses from dendritic spine loss and upregulated synaptic protein expressions in 3×Tg-AD mice. Proteomic analysis of 3×Tg-AD mouse hippocampal and cortical tissues showed that TBN induced neuroprotective effects through modulating mitophagy, MAPK and mTOR pathways. In particular, TBN significantly upregulated PINK1, a key protein for mitochondrial homeostasis, implicating PINK1 as a potential therapeutic target for AD. In summary, TBN improved cognitive functions in AD-related mouse model, inhibited Aβ production and tau hyperphosphorylation, and rescued synaptic loss and neuronal damage. Multiple mechanisms underlie the anti-AD effects of TBN including the modulation of APP processing, mTOR signaling and PINK1-related mitophagy.

## 1 Introduction

Alzheimer’s disease (AD) is the most common type of neurodegenerative disorder. During the past decades, remarkable progresses have been made in understanding the pathophysiology of AD, recognizing the multifactorial nature of AD and its relationship with inherited and environmental factors (Jeremic, Jimenez-Diaz, & Navarro-Lopez, 2021). The earliest phase of AD is the cellular phase when alterations in neurons, microglia, and astroglia induce subtle but perilous changes in the brain before cognitive impairment is observed (De Strooper & Karran, 2016). Many hypotheses have been put forward for the pathogenesis of AD, including amyloid-beta (Aβ) cascade, tau pathology, mitochondrial cascade, cholinergic neuron damage, glutamate excitotoxicity, oxidative stress, and neuroinflammation (Ali, 2016; X; Du, Wang, & Geng, 2018). Aβ deposition and neurofibrillary tangles (NFTs) of hyperphosphorylated tau in the brain is still widely accepted disease mechanism. These cellular and molecular alterations happen in parallel with accumulating Aβ followed by the spread of tau pathology associated with necroptosis in neurons (Scheltens et al., 2021). Extracellular accumulation Aβ as senile plaques and intraneuronal NFTs together with synaptic failure and neuronal loss are the main pathological features of AD (Hane, Lee, & Leonenko, 2017). Clinical evidence and experimental studies indicate an imbalance between Aβ production and clearance as the upstream event of Aβ dysregulation, which plays a critical role in AD onset, and leads to the formation of neurofibrillary tangles of tau protein, vascular damage, and neuronal loss (Selkoe & Hardy, 2016; Jeremic et al., 2021). Based on the Aβ hypotheses, numerous efforts have been undertaken to develop anti-AD therapeutic treatments (Tolar, Abushakra, & Sabbagh, 2020). Aducanumab is the first drug approved by the Food and Drug Administration (FDA) as a potential disease-modifying agent for mild AD, which has been shown to lower Aβ plaques in the brain (Esang & Gupta, 2021). The approval was not without controversy, due to utilizing Aβ plaque reduction as the surrogate endpoint. FDA required Biogen to continue phase four trials and provide evidence of clinical efficacy as predicted by the surrogate endpoint (Esang & Gupta, 2021). Therefore, potent disease-modifying treatment for AD is still lacking, and it is now established that anti-AD pharmacological treatments and multi-domain lifestyle interventions together could contribute to cognitive benefits in patients with increased risk of dementia (Scheltens et al., 2021).

The molecular alterations of AD pathophysiology apart from Aβ and NTFs are complicated and yet to be fully elucidated. Recent advances in molecular medicine such as large-scale multi-center study on AD human brain tissues by using quantitative mass spectrometry-based proteomics have provided invaluable data for AD research (Johnson et al., 2020). Unbiased, the mass spectrometry-based proteomic approach offers an efficient and comprehensive tool to understand biological network, pathway, and cell type changes in progression of AD, *via* quantitatively examine thousands of proteins at once using microscopic amounts of AD brain tissue (Thomas, 2019). Over the past decade, advances in proteomic studies have markedly accelerated the discovery of potential molecular mechanisms and cellular signaling pathways associated with AD pathology (Butterfield et al., 2003). Drummond et al. (2017) used a localized proteomics approach to study the proteome of amyloid plaques and NFTs, showing many novel proteins that had not previously been associated with AD, such as secerning-1, POTEE, ACTBM in Aβ plaques (Scheltens et al., 2021). Vega et al. (2018) reported that increased Ezrin protein expression was associated with early stages of neurodegeneration in tauopathy models and human disease. Volgyi et al. (2018) isolated the subcellular compartment mitochondrial-associated ER membrane (MAM) from the cerebral cortex of APP/PS1 mouse for proteomic analysis and demonstrated MAM protein changes are strongly associated with AD, particularly the ATP-binding cassette G1 (Abcg 1) which is a crucial protein for suppressing Aβ accumulation was highly and significantly changed in AD. Li et al. (2017) used proteomic approach to examine the early molecular events in hippocampus of APP_Swe/Ind_ mice, and revealed *ß*-spectrin and Rab-3a were key players in regulating APP processing in the early stage of AD mouse model. Another proteomic study with brain tissues from 12-month-old 3×Tg-AD mice revealed the reduced O-GlcNAcylation of several key proteins involved in important brain functions such as glucose metabolism and protein degradation (Tramutola et al., 2018). These findings have provided excellent evidence and resource for future targeted studies and will help discover novel therapeutic targets for AD (Thomas, 2019). Mass-spectrometry-based proteomics will continue to play an important role for drug discovery and biomarker development.

Tetramethylpyrazine nitrone (TBN) is a novel tetramethylpyrazine derivative with a nitrone moiety and displays potent free radical-scavenging activity. Tetramethylpyrazine is one of the main active ingredients of the herb medicine *Ligusticum wallichii Franchat* (Chuanxiong). We previously demonstrated that TBN exhibited potent free radical scavenging effects, protected rats from ischemic stroke damage and inhibited primary cortical neuronal death induced by glutamate and oxygen/glucose deprivation (OGD) *via* inhibiting calcium overload and maintaining mitochondrial function (G et al., 2018). TBN produced neuroprotective effects in chronic cerebral hypoperfusion rat model and primary hippocampal neurons exposed to hypoxia *via* regulating Bax/Bcl-2 and activating PI3K/Akt/p-GSK3β survival pathway (G et al., 2018). Despite being in different clinical classifications, AD and ischemic stroke share similar pathophysiological characteristics such as disrupted functions of the neurovascular unit and increased level of immune activation. Based on previous study, we further evaluated the neuroprotective and anti-AD effects of TBN in triple transgenic mouse models (3×Tg-AD) *in vivo* and the potential molecular underlying mechanisms in AD neuronal cell model (N2a/APPswe) *in vitro*. We further analyzed the hippocampal and cortical tissues from the mice by bioinformatics and quantitative proteomic analysis.

## 2 Materials and methods

### 2.1 Chemicals and reagents

TBN (purity >99%), donepezil (purity >99%) and memantine (purity >99%) were provided by Guangzhou Magpie Pharmaceuticals CO., LTD (Guangzhou, China); DMEM, FBS, penicillin, streptomycin, G418, BCA Kit, Pierce ECL Western Blotting substrate, Pierce high pH reversed-phase peptide fractionation kit and Sixplex TMT were purchased from Thermo Fisher Scientific (NJ, United States); Human Amyloid beta quantikine ELISA Kits used in this study were obtained from R and D systems (Minneapolis, MN, United States); FD Rapid GolgiStain^TM^ Kit was purchased from FD Neuro Technologies, Inc. (Columbia, MD, United States); Trypsin/Lys-C Mix was provided by Promega (Madison, Wisconsin, United States); Oasis HLB reversed-phase column chromatography was purchased from Waters corporation (MC, United States). Primary antibody 6E10 was purchased from Biolegend (San Diego, United States); Antibody D3D2N, p-S396tau, CADH4, SOD1, ATG3, VAMP3, CRTC1, LST8, RRAGC, *ß*-actin, and HRP-linked secondary antibody used in this study were obtained from Cell Signaling Technology (Beverly, MA, United States); Antibody APP, BACE1, PS1, ADAM10, IDE, NEP, Tau-5, synapsin I, synapsin II, PSD 95, PINK1 and Goat anti-mouse IgG H + L (Alexa Fluor^®^ 488) were purchased from Abcam (Cambridge, MA, United States). Antibody Tau-1was purchased from Merck (Darmstadt, Germany). CTF α/β antibody was purchased from Thermo Fisher Scientific (United States). Antibody sAPPα and sAPPβ were provided by IBL (Minneapolis, MN, United States).

### 2.2 Cell culture

Mouse wild-type Neuro-2a cell (N2a/WT) and Neuro-2a harboring human APP Swedish mutant cell (N2a/APPswe) were provided by Dr. Xifei Yang’ lab (Key Laboratory of Modern Toxicology of Shenzhen, Shenzhen Center for Disease Control and Prevention, Shenzhen, China). N2a/WT cells and N2a/APPswe cells were maintained in DMEM containing 10% FBS, 100 IU/mL penicillin-streptomycin, or 200 μg/mL G418 in humidified incubator with 95% air and 5% CO_2_ at 37°C. The cells were seeded in 25 cm^2^ culture flasks and treated with 300 μM TBN for 24 h, then cells were collected for further assay.

### 2.3 Animal care

Female 3×Tg-AD mice (APPswe, PSEN1M146V, and MAPTP301L transgenes) were provided by the Jackson Laboratory. The age-matched wild-type mice (female B6129SF2/J mice) (WT mice) were purchased from Guangdong Medical Laboratory Animal Center (Guangdong, China). The local reproduce was kept at the Shenzhen Center for Disease Control and Prevention (Shenzhen, China). The mice were kept with a 12 h artificial light/dark cycle (dark on 19:00–07:00) and able to get food and tap water freely. Eight-month old female 3×Tg-AD mice (12 mice at least in each group) were treated with TBN (60 mg/kg, i. g., bid), donepezil (1.3 mg/kg, i. g., qd), memantine (5 mg/kg, i. g., bid) or saline by gastric gavage for 4 months. The time gap between two administration was 8 h (9 a.m. and 5 p.m.). Eight-month old female WT mice were received with parallel volume of saline as a control group. After behavioral tests, mice were sacrificed with injection anesthetics (Zoletil 50/xylazine, ip), and brain tissues were collected for biochemistry analysis or proteomics analysis. All procedures were approved and supervised by the Institutional Animal Care and Use Committee of Shenzhen Center for Disease Control and Prevention.

### 2.4 Behavioral tests

Behavioral tests were conducted in 2 weeks before the end of drug treatment. The step-down passive avoidance (SDA) test, novel object recognition (NOR) test and Morris Water Maze (MWM) test were performed according to the protocol described in previous study (Zhou et al., 2019a; Zhou et al., 2019b). Briefly, SDA was performed in two stages: in a training trial, mice were gently placed on the grid floor while a foot shock was triggered (36 V) for 5 min. The test trial was performed 24 h after training trial, mice were gently put on the wooden platform, at the same time as electrical shock was delivered (36 V) for 5 min. The duration before mouse touching the grid floor at first time was recorded as the step-down latency, and the number of times that the mouse touching the grid floor with paws was recorded as the error times. The mice were allowed to have a rest for 3 days after SDA test, and then NOR test was initiated. Subsequently, NOR test was conducted. Briefly, the video-tracking system was operated to record the exploration activities when the mice were placed in plastic box which containing two identical objects (A+ A) or different objects (A + B). The discrimination index (DI) was calculated based on this equation (Time exploring object B- Time exploring object A)/(Time exploring object B+ Time exploring object A) × 100. After NOR test, another 3 days were also allowed before MWM test.

Finally, MWM test was performed after NOR test. Briefly, MWM test was performed in two stages: in training period, the mice had to explore the platform for five consecutive days, four times per day. The spending time for mice to locate the submerged platform at first time was defined as the escape latency. Probe test was executed on the sixth day after training period, during which the platform was removed, and the mice were allowed to swim freely for 2 min. The time spent for mice to cross the platform site at first time was defined as the latency to cross platform location. During MWM test, movement-tracking system (Supermaze^+^) was operated to record the swimming trajectory.

### 2.5 Quantification of soluble and insoluble Aβ by using ELISA

Aβ was detected according to the protocol described in our previous study (Zhou et al., 2019a; Zhou et al., 2019b). Briefly, hippocampus or cerebral cortex tissue was harvested and homogenized in RIPA with phosphatase and protease inhibitor. The supernatant fraction was collected as soluble Aβ after centrifugation (20,000 × *g*, 30 min, 4°C). The precipitation was collected as the insoluble Aβ, and resuspended with 70% formic acid. The levels of A*β*_1-40_ and A*β*_1-42_ were measured using a Quantikine ELISA Human Amyloid *ß* (aa_1-40_/aa_1-42_) Immunoassay kits.

### 2.6 Immunohistochemistry

The deposition of Aβ plaques in subiculum of hippcampus was measured using 6 E10 antibody by Immunohistochemical (IHC) staining. Briefly, dissected brain tissues were fixed with 4% paraformaldehyde for 24 h at room temperature, and then embedded in paraffin block. Paraffin block were sectioned at 8–10 μm thickness on a microtome and transferred onto glass slides suitable for IHC stain. Slides were treated with xylene for 2 times and gradual rehydration with ethanol (100%, 95%, 70%, 50%) respectively for 3 min each. After blocking endogenous peroxidase activity and performing antigen retrival, slides were incubated with 10% fetal bovine serum in PBS for 1 h at room temperature. Slides were then incubated with diluted primary antibody (6 E10, 1:2000) overnight at 4°C, and then incubated with secondary antibody (HRP-goat anti-mouse IgG, 1:5,000) in the dark for 1 h at room temperature. The immunoreaction was visualized with the diaminobenzidine tetrachloride system. The images were observed using a microscope at ×40 magnification (Olympus BX41, Tokyo, Japan).

### 2.7 Golgi staining

Golgi staining was performed as previously report (Zhou et al., 2019a; Zhou et al., 2019b). Briefly, brain tissues were dipped into impregnation solution for 1 week, and the staining procedures was conducted according to the instruction of FD Rapid GolgiStain^TM^ Kit. The neuros structure were observed under confocal laser scanning microscope at ×60 magnification (Leica TCS SP5), and the number of dendrites spine in the hippocampus neuros were calculated using LAS AF Lit software.

### 2.8 Western blot

Hippocampus tissues were lysed with RIPA buffer containing 1% protease inhibitor, and the protein were separated by SDS–PAGE gel. After protein transferred into 0.2 μm polyvinylidene difluoride membranes, the membranes were blocked with 5% defatted milk for 1 h. The membranes were incubated with primary antibodies such as APP, BACE1, Tau, *ß*-actin at 4°C overnight, and then incubated with secondary antibody conjugated with horseradish peroxidase. The blots were developed with Pierce ECL Western.

Blotting substrate (United States) with Quantity one software (Bio-Rad).

### 2.9 Proteomics analysis

Proteomics analysis was performed according to the protocol described in our previous study (Zhou et al., 2019a; Zhou et al., 2019b). The procedure including sample preparation, Tandem mass Tag (TMT) labeling, NanoLC-ESI-MS/MS analysis and bioinformatics analysis. In briefly, a total of 100 mg protein sample (6 mice in each experimental group, 16.7 μg of protein from per mouse) extracted from hippocampus or cerebral cortex was digested with trypsin/Lys-C (Promega, WI, United States), and then acidified with pure formic acid (FA) to achieve supernatant (final pH 1–2). Subsequently, the protein samples were labeled with TMT isobaric (Thermo Scientific, NJ, United States) after desalted, and then divided into 8 fractions by high pH reversed-phase peptide fractionation kit (Thermo Scientific, NJ, United States). The fractions was dried to achieve the lyophilized peptide using a vacuum centrifuge. The peptides were resuspended in loading buffer (0.1% formic acid with 2% acetonitrile), and then loaded on ChromXP C18 trap column to process the high performance liquid chromatography (Eksigent nanoLC-Ultra™ 2D system) and mass spectrometry (SCIEX, Concord, ON). Raw data were converted to mgf peaklist with PEAKS 8.5 software (Bioinformatics Solutions, Waterloo, Canada), and searched against UniProt-Mus musculus database (released in July 2017). The ratios of altered proteins in each group were set as group AD/WT, group TBN/WT and group TBN/AD. The ratio of TBN/AD were considered to be the fold change. It was defined as significant upregulation or downregulation when fold change≥1.2 or <0.83. Bioinformatics analysis were performed by DAVID Bioinformatics resources 6.8 and Cytoscape (3.6.0). The mass spectrometry proteomics data have been deposited to the ProteomeXchange Consortium *via* the PRIDE partner repository with the dataset identifier PXD039514.

### 2.10 Graphing and statistical analysis

Statistics were performed by GraphPad Prism 7.0. All data were presented as mean ± SEM, and the *p*-value were analyzed by using one-way ANOVA or two-way ANOVA followed by Tukey’s multiple comparison test. *p*-value lower than 0.05 was considered statistically significant.

## 3 Results

### 3.1 TBN reverses AD phenotype in N2a/APPswe cell model

**FIGURE 1:**
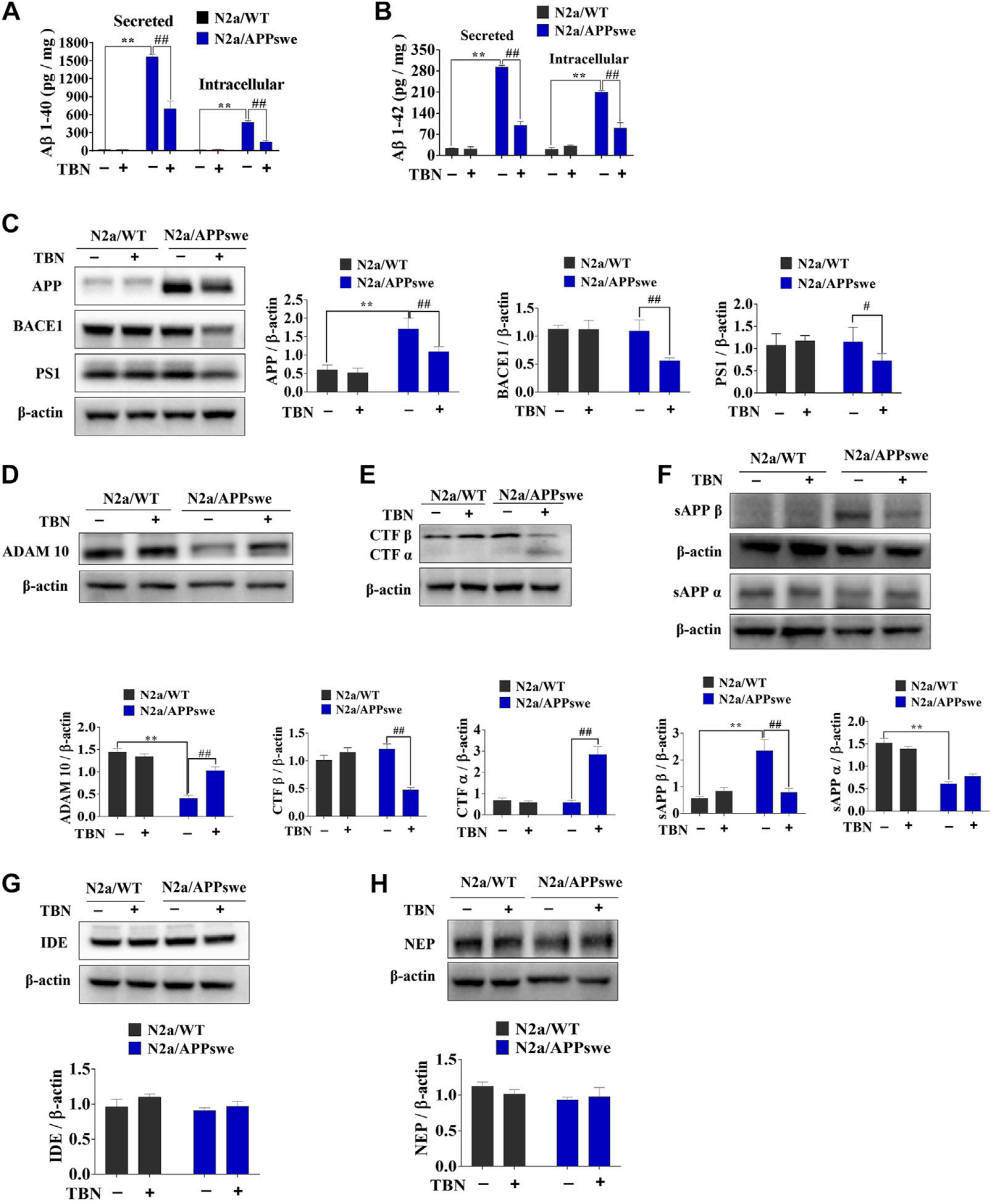
TBN treatment inhibits amyloidogenic APP processing and promotes nonamyloidgenic APP processing in N2a/APPswe cells. The cell culture medium or cell lysates were collected after 24 h treatment with TBN (300 μμM). **(A B)** The level of the secreted and intracellular Aβ40/42 in N2a/WT and N2a/APPswe cells were measured by enzyme-linked immunosorbent assay (ELIAS). **(C)** Representative Western blots of APP, BACE1, PS1 and ß-actin in the N2a/WT and N2a/APPswe treated with or without TBN. **(D–F)** Representative Western blots of ADAM 10, CTF α, CTFβ, sAPPα, sAPPβ and ß-actin in the N2a/WT and N2a/APPswe treated with or without TBN. **(G,H)** Representative Western blots of IDE and NEP and ß-actin in the N2a/WT and N2a/APPswe treated with or without TBN. Values represent mean ± SEM, n ≥ 3, **p <0.01 vs N2a/WT Ctrl, ^#^ *p* < 0.05 or ^##^ *p* < 0.01 vs. N2a/APPswe Ctrl, two-way ANOVA.

Extracellular Aβ accumulation and intracellular tau hyperphosphorylation are characteristic neuronal phenotype of AD. Aβ is generated intracellularly from the sequential cleavages of APP by *ß*-secretase and *?* secretase complex. Alternatively, APP can be cleaved by *a* secretase to produce sAPPα which prevents Aβ generation. By employing N2a cells stably expressing human APP695 (N2a/APPswe), we first examined the effects of TBN on the soluble Aβ levels of intracellular and secreted Aβ by using sandwich ELISA. As shown in Figures 1A, B, TBN significantly reduced the levels of both secreted and intracellular Aβ40 and Aβ42 in N2a/APPswe cells, while having no effect on Aβ levels in the control cell type (N2a/WT). We further examined the expression levels of crucial proteins in the APP processing pathways by Western blot. When compared with N2a/WT, it was observed the levels of total APP was significantly elevated (Figure 1C) while ADAM10 (α secretase) was markedly reduced in N2a/APPswe (Figure 1D), but the levels of BACE1 (β secretase) and PS1 (major component of γ secretase) were similar in both cell types (Figure 1C). TBN treatment was effective at suppressing the increased level of APP, promoting the reduced level of ADAM 10, downregulating the BACE1 and PS1 and reducing Aβ production in N2a/APPswe (Figures 1A–D). We further evaluated downstream proteins of the APP processing and observed increased sAPPβ level and attenuated sAPPα level in N2a/APPswe (Figure 1F), but the levels of CTFβ and CTFα were similar in both cell types (Figure 1E). TBN treatment could decrease the sAPPβ and CTFβ levels, and increase sAPPα and CTFα levels.

We further evaluated the protein expressions of neprilysin (NEP) and insulin-degrading enzyme (IDE) (two major proteases involved in Aβ degradation) and showed that their expression levels were similar in both N2a/APPswe and N2a/WT. TBN had negligible effects on the expressions of IDE and NEP (Figure 1G, H).

**FIGURE 2:**
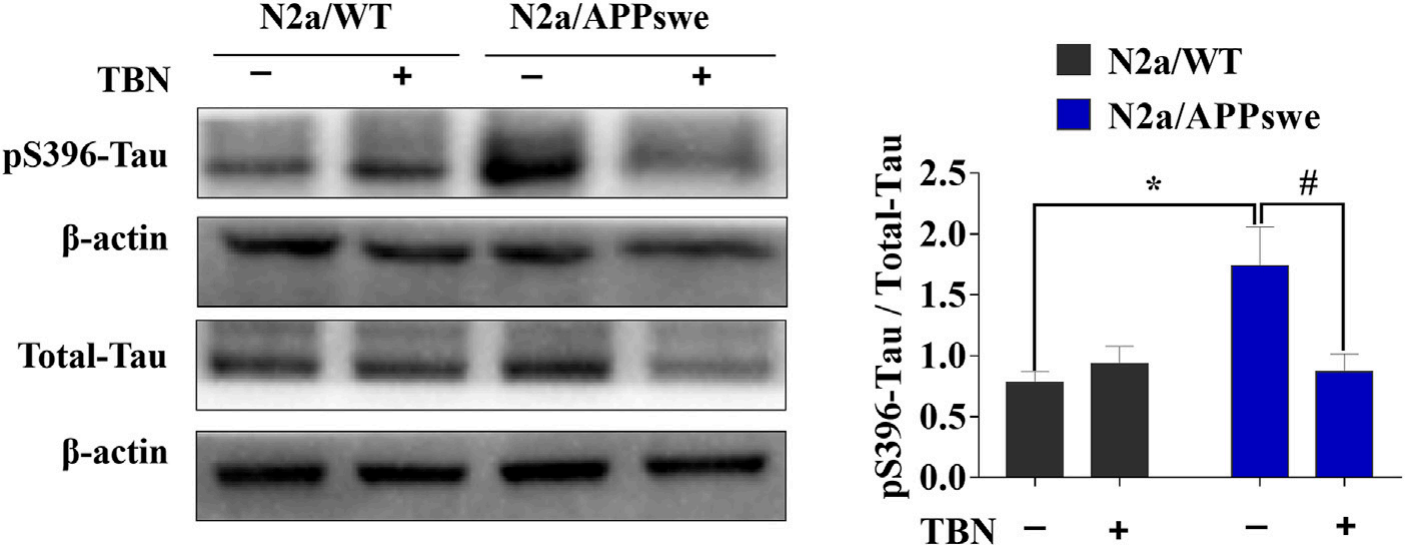
TBN treatment attenuates tau phosphorylation in N2a/APPswe. The levels of phosphorylated tau (pS396), total tau and *ß*-actin were determined by Western blotting. Values represent mean ± SEM, n = 4, *p < 0.05 vs. N2a/WT Ctrl, ^#^ *p* < 0.05 vs. N2a/APPswe Ctrl, two-way ANOVA.

Next, we examined whether TBN could impact tau phosphorylation in N2a/APPswe by using antibodies against phosphorylated-tau at serine 396 (pS396) and total tau (Tau-5) analyzed by Western blot. As shown in Figure 2, elevated pS396-tau level was observed in N2a/APPswe, which was significantly reduced by TBN treatment. In addition, TBN also markedly reduced total tau level in N2a/APPswe but not in N2a/WT. Taken together, TBN effectively reversed the AD phenotype in N2a/APPswe cells.

### 3.2 TBN improves behavioral performance in transgenic mouse model of AD

**FIGURE 3:**
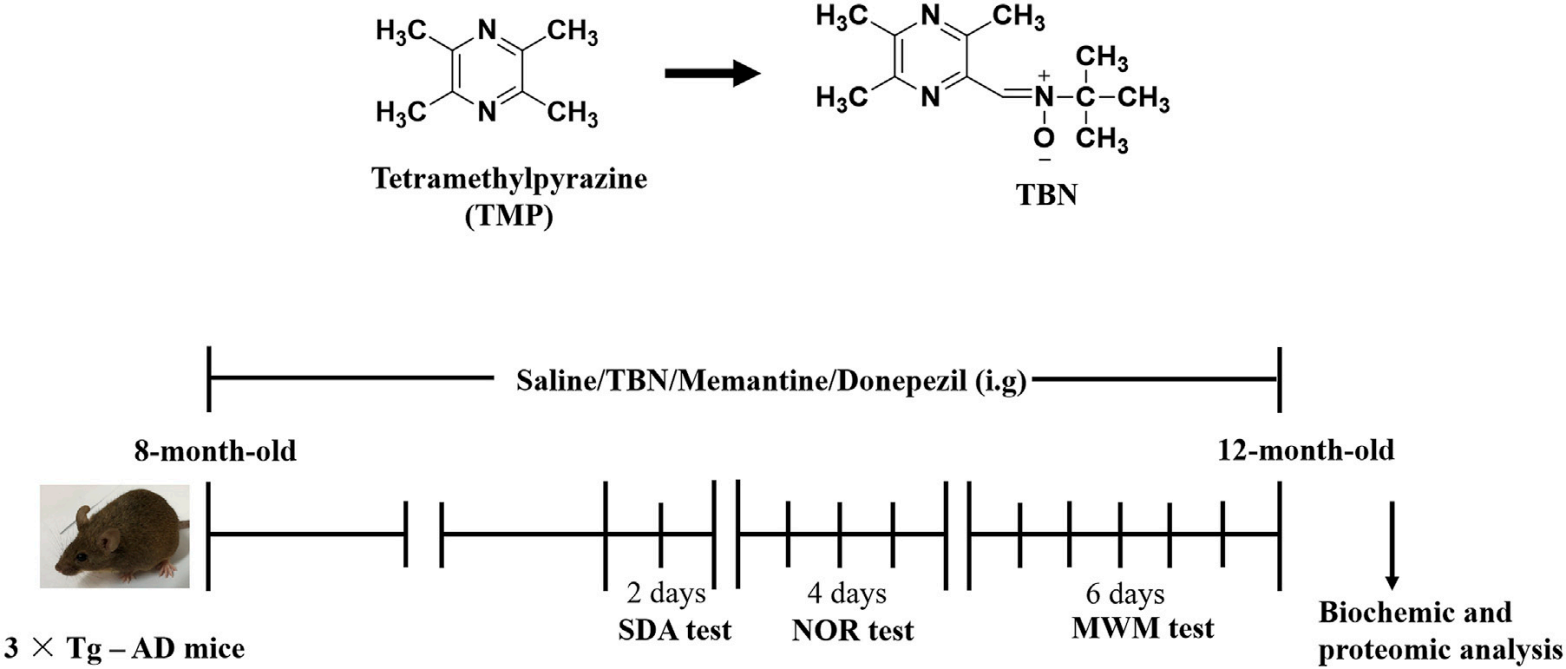
Chemical structures and experimental design for animal model. The chemical structures of TMP and TBN. The process design of TBN treatment study in 3×Tg-AD mice. SDA test: step-down avoidance (SDA) test. NOR test: novel object recognition (NOR) test. MWM test: morris water maze (MWM) test.

**FIGURE 4:**
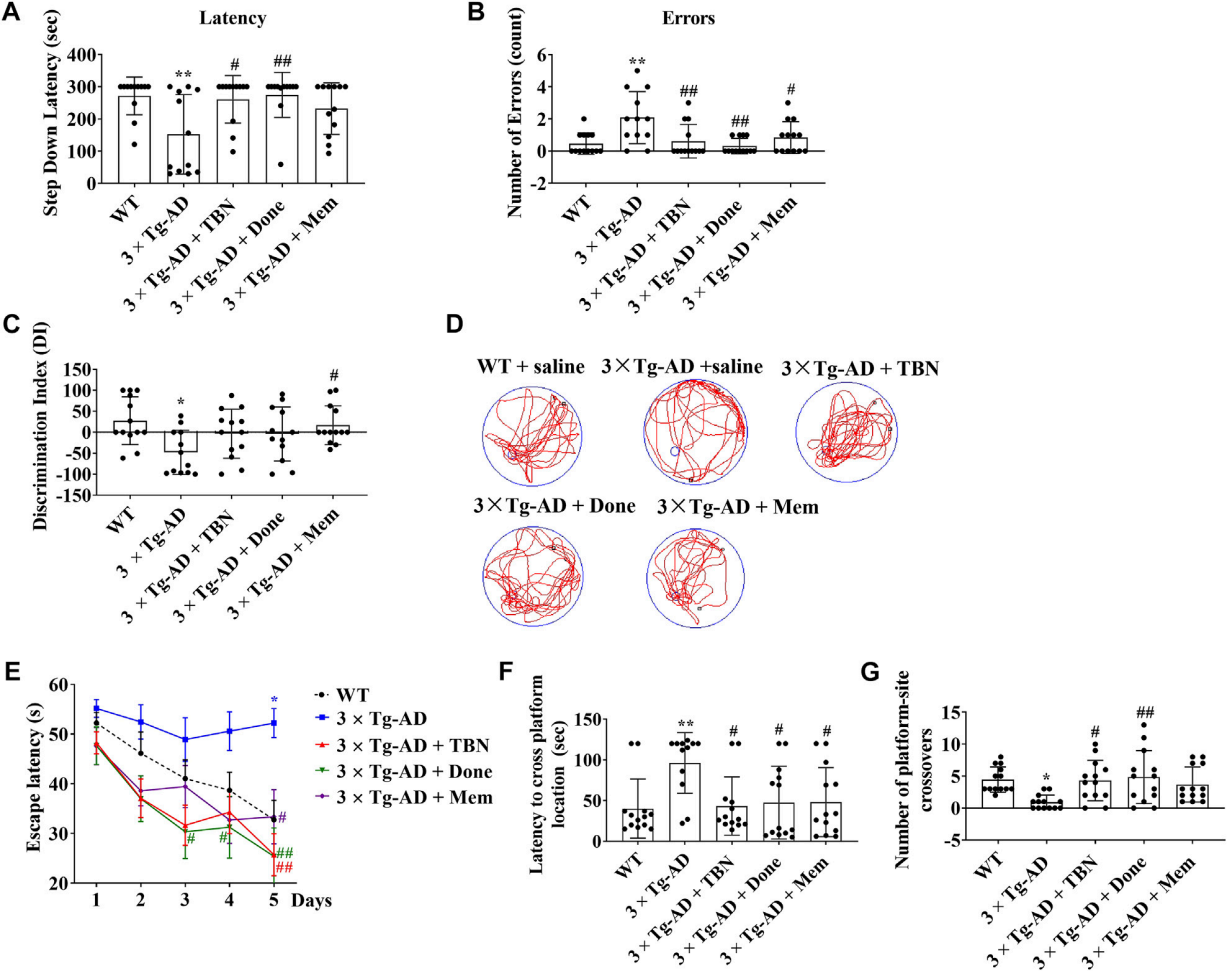
TBN improved cognitive capabilities of 3×Tg-AD mice. **(A, B)** TBN (60 mg/kg) significantly increased the step-down latency in memory retention test and decreased the number of errors made by 3×Tg-AD mice in SDA test. Donepezil and Memantine as the positive drugs. Done: Donepezil; Mem: Memantine. **(C)** TBN improved the recognition memory as shown by an increased discrimination index in 3×Tg-AD mice in NOR test. **(D–G)** TBN improved spatial memory and cognitive deficits of 3×Tg-AD mice in MWM test. **(D)** Representative swim traces of mice in the MWM after removing the platform during probe test. **(E)** Escape latency of mice to reach the platform during training (Day 1–5). **(F)** The latency of mice to cross from starting position to target quadrant during probe test. **(G)** Number of annulus crossing in target quadrant during probe test. Data were presented as mean ± SD, n ≥ 12, *p < 0.05 or **p < 0.01 vs. WT group, ^#^ *p* < 0.05 or ^##^ *p* < 0.01 vs. 3×Tg-AD group, one-way ANOVA.

The procedure of animal experiment was showed in Figure 3. We evaluated the protective effects of TBN on cognitive impairment in 3×Tg-AD mice. Animals were administered with TBN (60 mg/kg) for 4 months (Figure 3). Compared with saline-treated 3×Tg-AD mice, TBN treatment performed better parameter in the SDA, NOR, and MWM tests, as reflected by a significant increased step-down latency and less errors (Figures 4A, B), improved discrimination index (DI) (Figure 4C) and number of crossings (Figure 4G), and a significant reduction in the escape latency (Figures 4E, F). The trial trace record (Figure 4D) showed that TBN treatment notably increased the explore trajectory in target quadrant compared with 3×Tg-AD mice. Taken together, these results indicate that TBN could prevent or halt cognitive dysfunction in AD mice.

### 3.3 TBN reduces Aβ levels and plaque deposition in transgenic mouse models of AD

**FIGURE 5:**
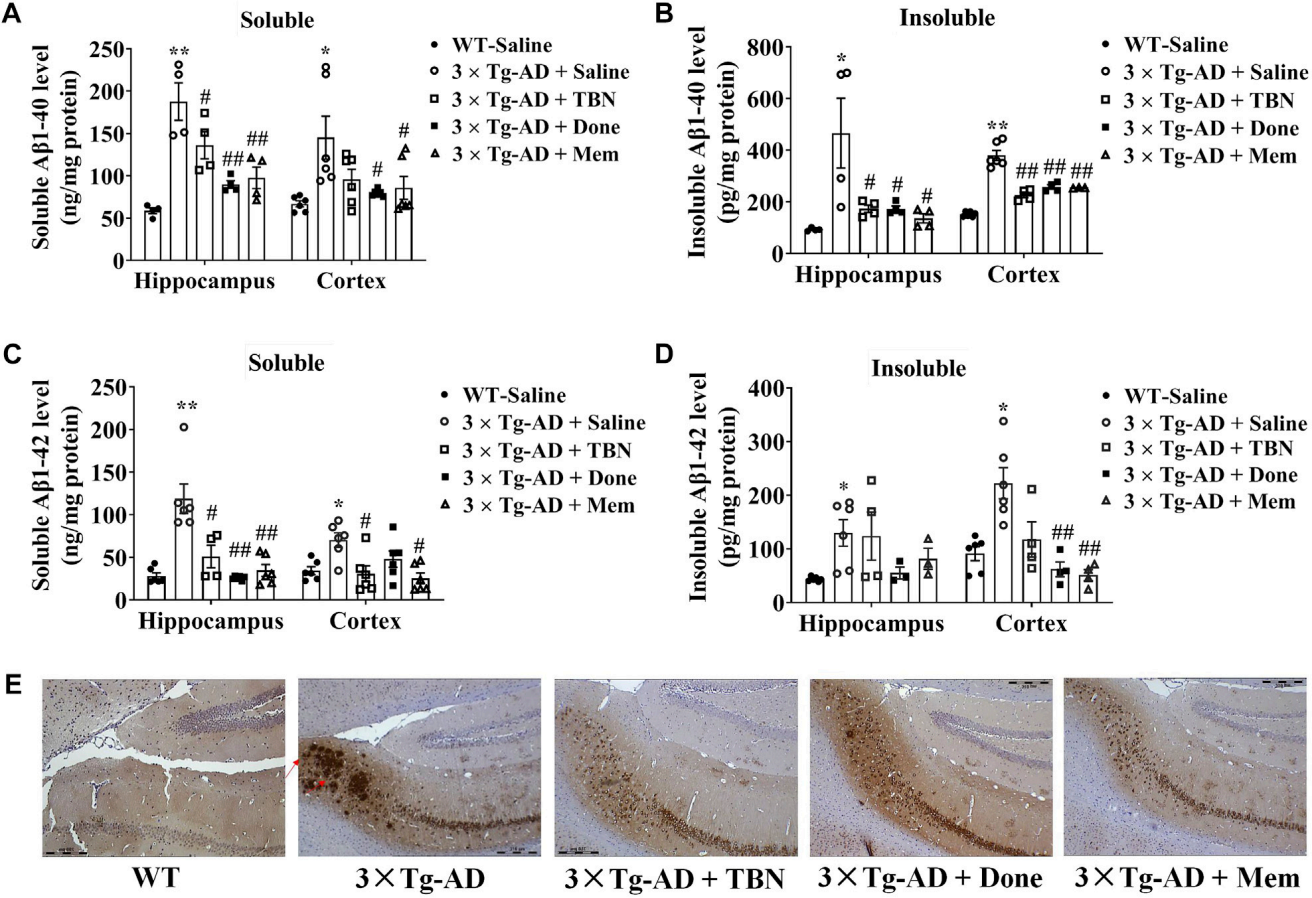
TBN treatment reduced the levels of Aβ in 3×Tg-AD mice. **(A–D)** Levels of soluble and insoluble Aβ40/Aβ42 in homogenates of hippocampal tissues and cerebral cortex of 3×Tg-AD mice or WT mice were determined by using ELISA. **(E)** Immunohistochemical staining by Aβ antibody (6E10) in the subiculum of hippocampal tissues. Data were presented as mean ± SEM, n ≥ 4, *p < 0.05 or **p < 0.01 vs. WT group, ^#^ *p* < 0.05 or ^##^ *p* < 0.01 vs. AD group, two-way ANOVA.

We evaluated the effects of TBN on Aβ deposition in 3×Tg-AD mice. It is reported 3×Tg-AD mice develop age-related and progressive neuropathology including plaques and tangles, with Aβ depositions starting at 6 months of age, and the plaque deposition becomes extensive and the tau pathology becomes evident by 12 months (Oddo et al., 2003). We observed TBN treatment reduced Aβ accumulation in the hippocampal and cortical tissues of 3×Tg-AD as evaluated by ELISA (Figures 5A–C). TBN significantly reduced both soluble and insoluble levels of Aβ40 and soluble Aβ42, while a downward trend was also observed for insoluble Aβ42 (Figure 5D). Immunohistochemical staining with anti-Aβ (6E10) antibody showed that TBN remarkably removed the deposition of Aβ plaques in the hippocampal subiculum (Figure 5E). Thus, these results indicate that TBN treatment could decrease the total levels of Aβ and plaque deposition in 3×Tg-AD mice.

### 3.4 TBN attenuates APP and PS1 expressions and tau hyperphosphorylation in the hippocampal tissues of transgenic mouse model of AD

**FIGURE 6:**
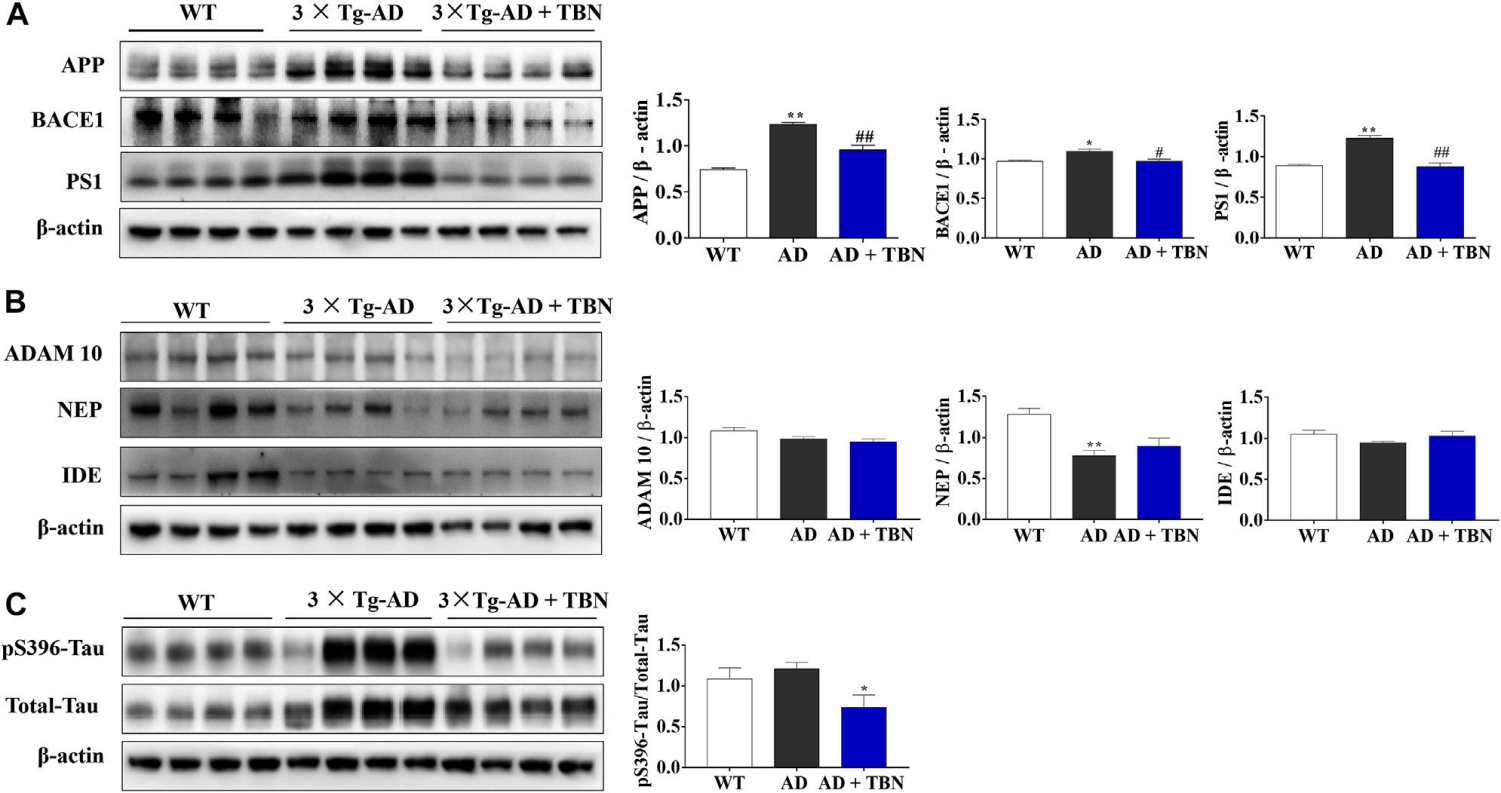
TBN treatment regulated APP processing and hyperphosphorylated tau in 3×Tg-AD mice. **(A)** Representative Western Blotting of APP, BACE1, PS1 and *ß*-actin in the hippocampal tissues of AD mice or WT mice. **(B)** Representative WB of ADAM10, NEP, IDE and *ß*-actin in the hippocampal tissues of AD mice or WT mice. **(C)** The levels of phosphorylated tau (pS396), total tau (Tau 5) and *ß*-actin were determined by Western blotting. Data were presented as mean ± SEM, n ≥ 4, *p < 0.05 or **p < 0.01 vs. WT group, ^#^ *p* < 0.05 or ^##^ *p* < 0.01 vs. AD group, one-way ANOVA.

As anticipated, there were upregulated protein levels of total APP and *?* secretase (PS1) in the hippocampal tissues of 3×Tg-AD mice, while the levels of *ß* secretase (BACE1) were slightly increased and no significant change in *a* secretase (ADAM 10) compared with WT mice (Figures 6A, B). TBN treatment significantly reversed the upregulation of APP, PS1 and BACE1, but had no effect on the level of ADAM 10. We further investigated if TBN affected Aβ clearance in 3×Tg-AD mice. The level of the Aβ-degrading enzyme NEP was markedly reduced in 3×Tg-AD mice, whereas IDE was not altered (Figure 6B). We observed a slight increase in NEP expression after TBN treatment, although it was not statistically significant (Figure 6B). Next, we measured the levels of total tau (Tau 5) and phosphorylated tau (pS396) by Western blot. Tau is typically hyperphosphorylated in 3×Tg-AD mice from age 12–15 months. We observed the levels of phosphorylated tau (pS396) and total tau (Tau-5) were significantly elevated when compared with WT animals, and were markedly reduced after TBN treatment (Figure 6C). Taken together, these data indicate that TBN produces its therapeutic benefits in 3×Tg-AD mice through effectively inhibiting the expressions of APP and PS1 and preventing the hyperphosphorylation of tau in the hippocampal tissues.

### 3.5 TBN restores synaptic function and dendritic spines in the hippocampal tissues of transgenic mouse models of AD

**FIGURE 7:**
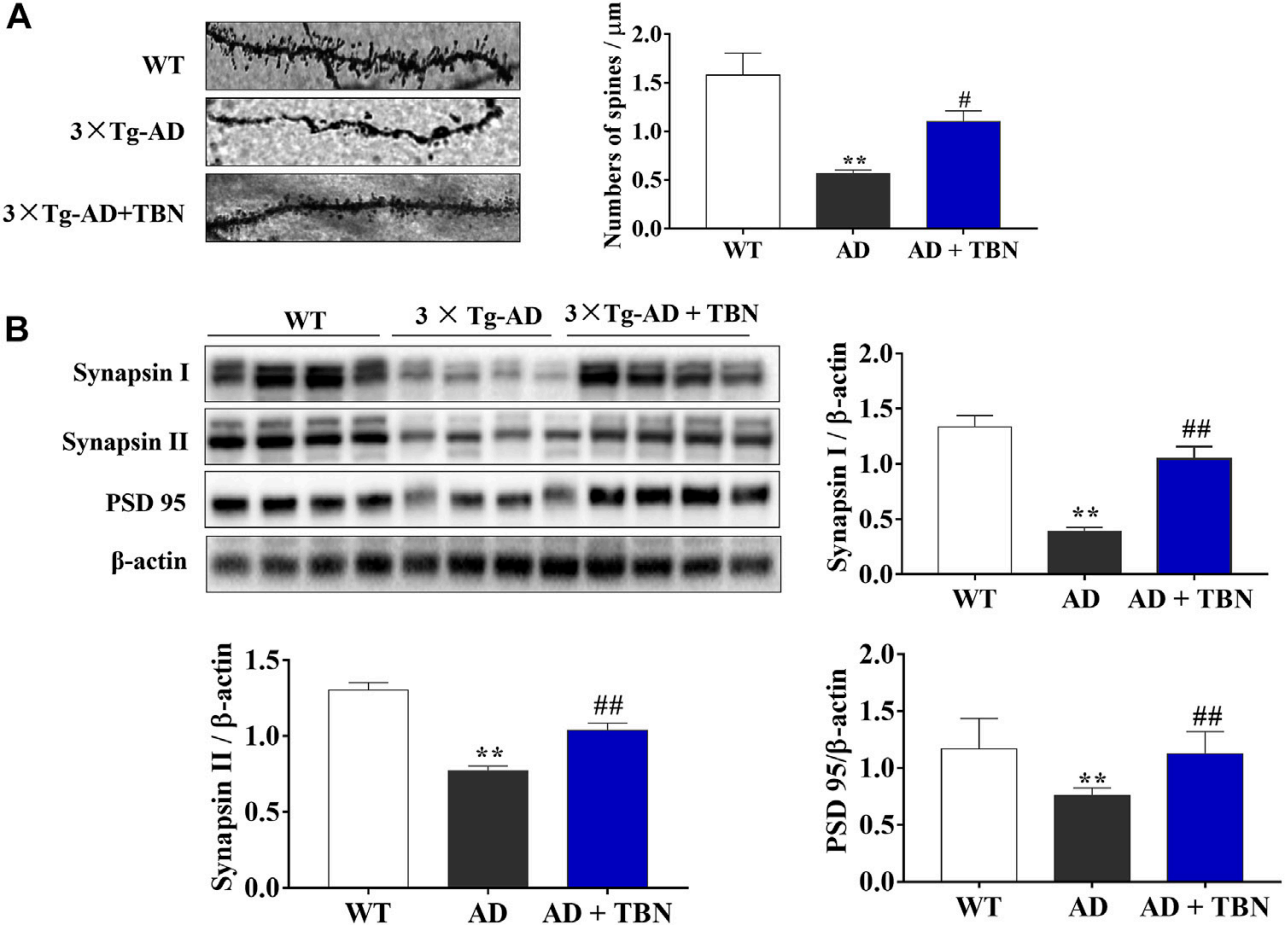
Effects of TBN on expression of synapse protein and dendritic spine loss. **(A)** Representative images of Golgi staining from hippocampal dendrites of 3×Tg-AD by confocal images (on the left). The number of dendritic spines is indicated within graph bars (on the right). **(B)** The levels of synapsin I, synapsin II and PSD 95 in the hippocampus of mice were measured by Western blotting. Data were presented as mean ± SEM, n ≥ 4. **p < 0.01 vs. WT group, ^#^ *p* < 0.05 or ^##^ *p* < 0.01 vs. 3×Tg-AD group, one-way ANOVA.

Synaptic plasticity is widely considered to be a pivotal mechanism in cognition dysfunction, and proteins involved in synaptic function and synaptogenesis progressively decrease throughout AD. To further investigate the neuroprotective effects of TBN, we determined the density of dendritic spines using Golgi staining in the hippocampus of 3×Tg-AD mice. The dendritic spines were markedly decreased in saline-treated 3×Tg-AD mice compared with WT mice (Figure 7A). More importantly, TBN treatment significantly reversed the reduction of dendritic spines. To further confirm these findings, we examined the levels of synapsin I, synapsin II and PSD95 proteins by Western blot. Saline-treated 3×Tg-AD mice showed remarkably reduction in these synapse-associated proteins, and more interestingly TBN treatment significantly increased the expression of these synaptic markers (Figure 7B). Taken together, these data indicated that TBN could against synaptic loss and improve the expression of synaptic proteins.

### 3.6 TBN remodels the hippocampal and cortical proteomes of 3×Tg-AD mice

**FIGURE 8:**
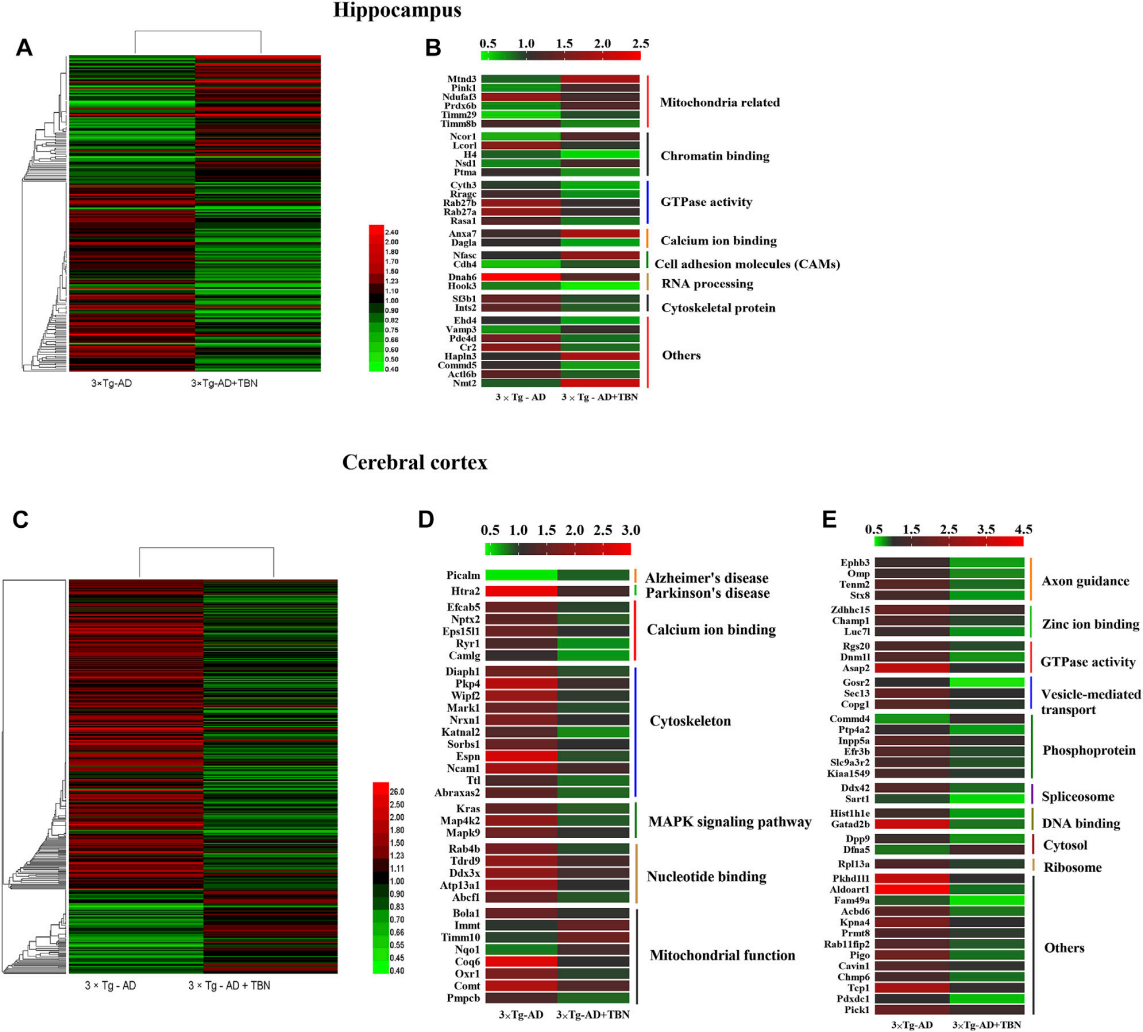
Heat mapping of altered proteins by TBN treatment in AD mice. **(A)** Hierarchical clustering of 210 changed proteins in the hippocampus between 3×Tg-AD group and TBN group. **(B)** 32 significantly altered proteins in the hippocampus related to mitochondrial, calcium ion binding, cytoskeleton and RNA processing between 3×Tg-AD group and TBN group. **(C)** Hierarchical clustering of 401 changed proteins in the cerebral cortex between 3×Tg-AD group and TBN group. **(D,E)** 74 significantly altered proteins in the cerebral cortex related to cytoskeleton, MAPK signaling pathway, mitochondrion function, axon guidance and DNA binding were identified between 3×Tg-AD group and TBN group. Color of each cell stand for degree of protein expression, red cell indicated rising level and green cell indicated reducing level compared with WT group. Heat mapping was performed by Heml 1.0.3.7-Heatmap Illustrator. Classification was performed by GraphPad Prism 7.0.

The hippocampal or cortical tissues of WT, 3×Tg-AD and TBN treatment 3×Tg-AD mice were analyzed by TMT labeling proteomic approach. In the hippocampus, 84 proteins were significantly upregulated and 126 proteins were significantly downregulated (fold change ≥1.2 or <0.83) (Figure 8A), where 32 of the proteins with dramatic up- or downregulation (fold change ≥1.5 or <0.67, Supplementary Table A-S1). These proteins were involved in mitochondrial function, GTPase activity and cytoskeleton organization (Figure 8B). In the cerebral cortex, 86 proteins were significantly upregulated and 315 proteins were significantly downregulated (fold change ≥1.2 or <0.83) (Figure 8C). Among them, 74 proteins dramatically changed in expression levels (fold change ≥1.5 or <0.67, Supplementary Table A-S2), which were related to calcium ion binding, cytoskeleton organization, MAPK signaling pathway, mitochondrial function, axon guidance and GTPase activity (Figure 8D, E). All these proteins with dramatic altered expression in the hippocampus and cortex are highly associated with AD pathogenesis.

**FIGURE 9:**
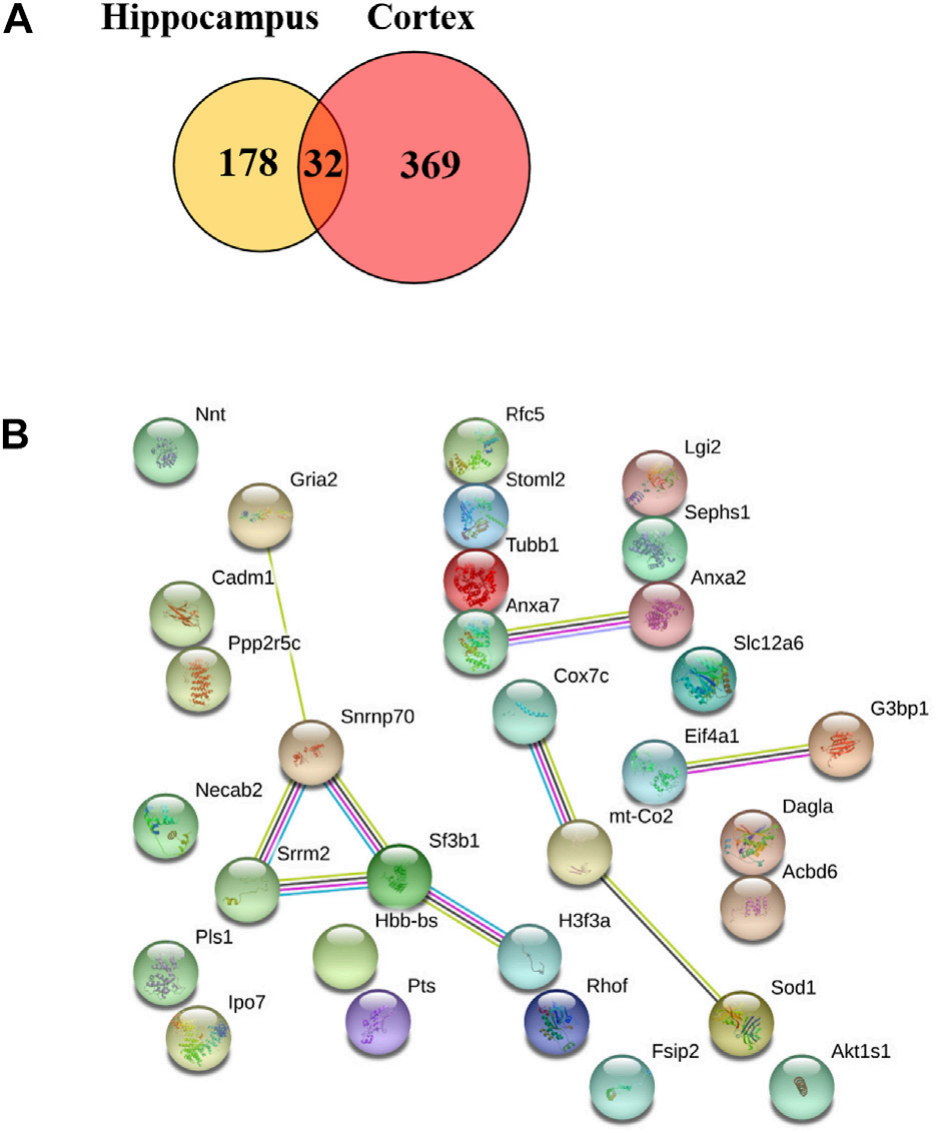
Venn diagram of two repeated analysis of the significant changed protein in the hippocampus and cerebral cortex. **(A)** The Venn diagram showing the overlap of differently expressed proteins after TBN treatment. **(B)** The PPI network of the 32 proteins.

We also found that 32 proteins were remarkably changed in both the hippocampus and the cerebral cortex (TBN/AD fold change ≥1.2 or <0.83, Supplementary Table A-S3) including ANXA 7, SODC, COX2 (mt-Co2), COX7C, GRIA2, SRRM2 and SF3B1 (Figure 9). However, some of the proteins under the effect of TBN showed contrary expression patterns. FSIP2 and DGLA were downregulated in the hippocampus but upregulated in the cerebral cortex. On the other hand, IPO7, RHOF, ANXA2, SPS1, RFC5, ACBD6 and RU17 were upregulated in the hippocampus whereas they were downregulated in the cortex.

### 3.7 Bioinformatics analysis of altered proteins by TBN treatment in 3×Tg-AD mice

**FIGURE 10:**
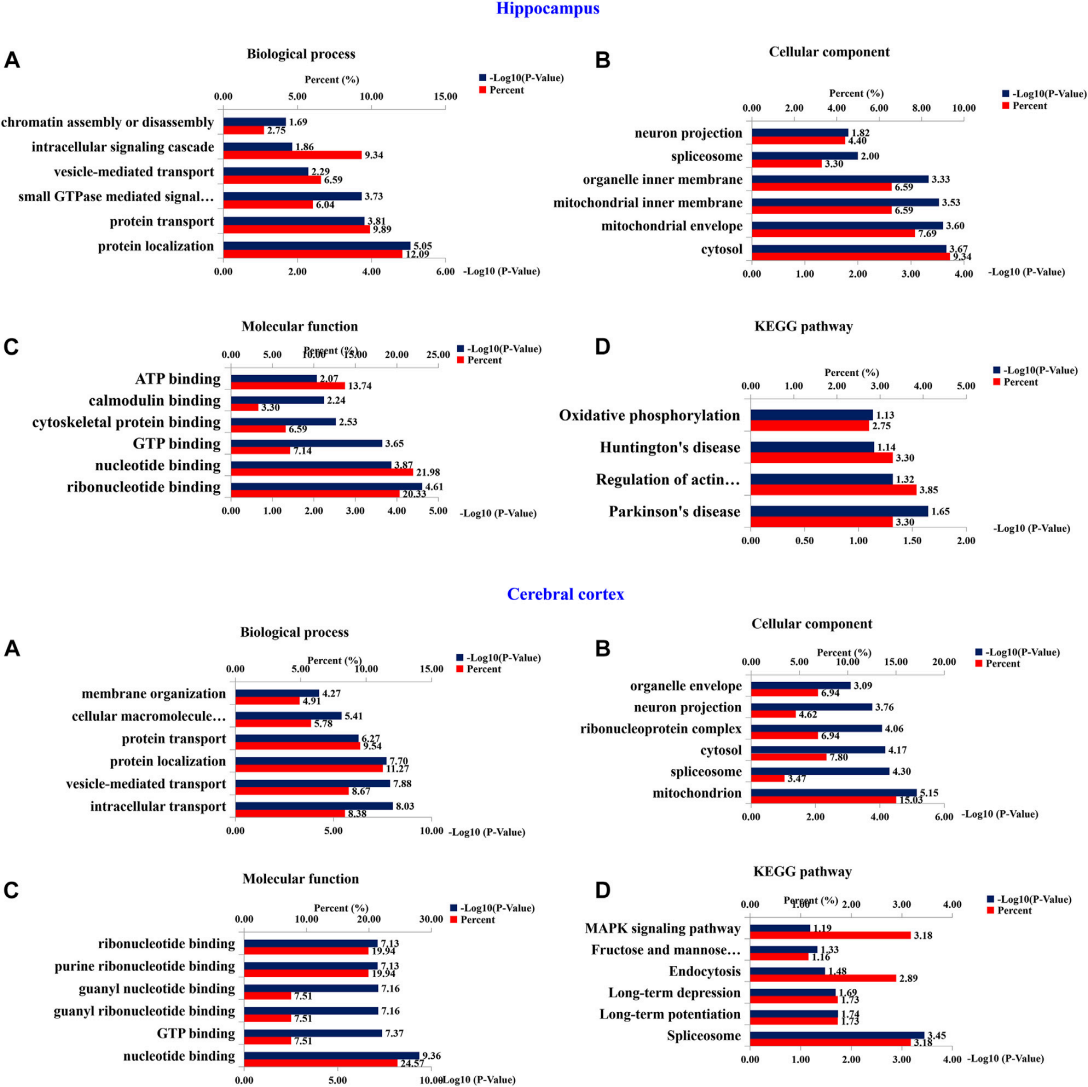
Bioinformatics analysis of differentially expressed proteins of hippocampus and cerebral cortex. 210 (Hippocampus AD) and 401 (Cerebral Cortex AD) changed proteins in the hippocampus and cerebral cortex, respectively, were analyzed by DAVID GO analysis and KEGG analysis. Proteins were functionally annotated in according to their biological process, cellular component and molecular function terms, and listed according to the –Log10 (*p*-value).

To investigate the functional significance of these altered proteins, functional classification (Gene Ontology terms) was performed by using DAVID bioinformatics resources (version 6.8). The results as shown in the Figure 10. The most representative terms of biological process in the hippocampus were protein localization, protein transport, vesicle-mediated transport and intracellular signaling cascade chromatin assembly or disassembly. Cytosol, mitochondrial envelope, mitochondrial inner membrane, organelle inner membrane, spliceosome and neuron projection were the most over-represented terms of cellular component in the hippocampal proteins. Ribonucleotide binding, nucleotide binding, GTP binding, cytoskeletal protein binding, calmodulin binding and ATP binding were the most enriched terms of molecular function were in the hippocampal proteins. In the cerebral cortical proteins, the most enriched terms of biological process were intracellular transport, vesicle-mediated transport, protein localization, protein transport, cellular macromolecule localization and establishment of membrane organization. The most representative terms of cellular component were mitochondrion, spliceosome, cytosol, ribonucleoprotein complex, organelle envelope, and neuron projection. The most enriched terms of molecular function were nucleotide binding, GTP binding, guanyl ribonucleotide binding, guanyl nucleotide binding, purine ribonucleotide binding and ribonucleotide binding. For hippocampal proteins, the highly represented terms of KEGG pathway included oxidative phosphorylation, Huntington’s disease, regulation of actin cytoskeleton and Parkinson’s disease. In the cerebral cortex, the highly enriched terms included MAPK signaling pathway, fructose and mannose metabolism, endocytosis, long-term depression, long-term potentiation and spliceosome.

**FIGURE 11:**
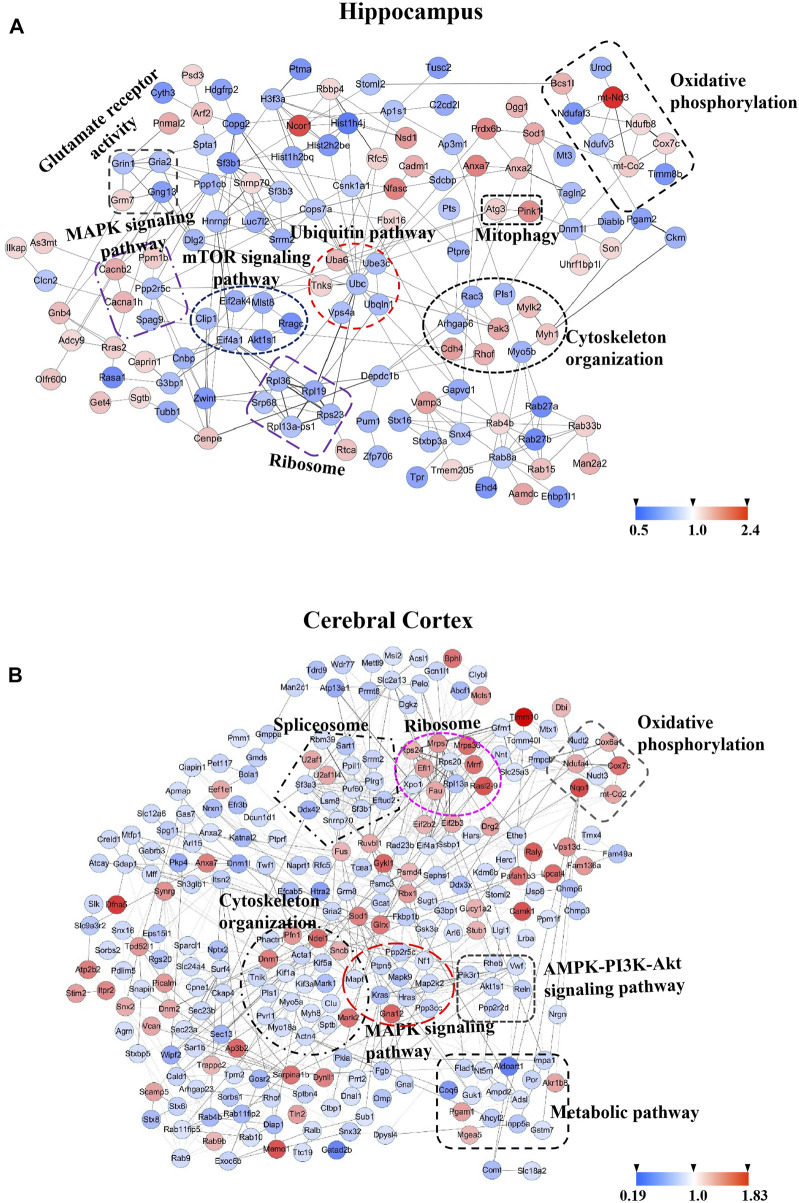
Protein-protein interaction (PPI) analysis of significantly changed proteins of hippocampus and cerebral cortex using STRING database and mapped by using Cytoscape 3.6.0. **(A)** PPI network of 210 differentially expressed proteins of hippocampus. **(B)** PPI network of 401 differentially expressed proteins of cerebral cortex. Circles indicate protein, gray lines indicate the interactions between two proteins, red node indicate upregulated proteins, and blue node indicate downregulated proteins.

**FIGURE 12:**
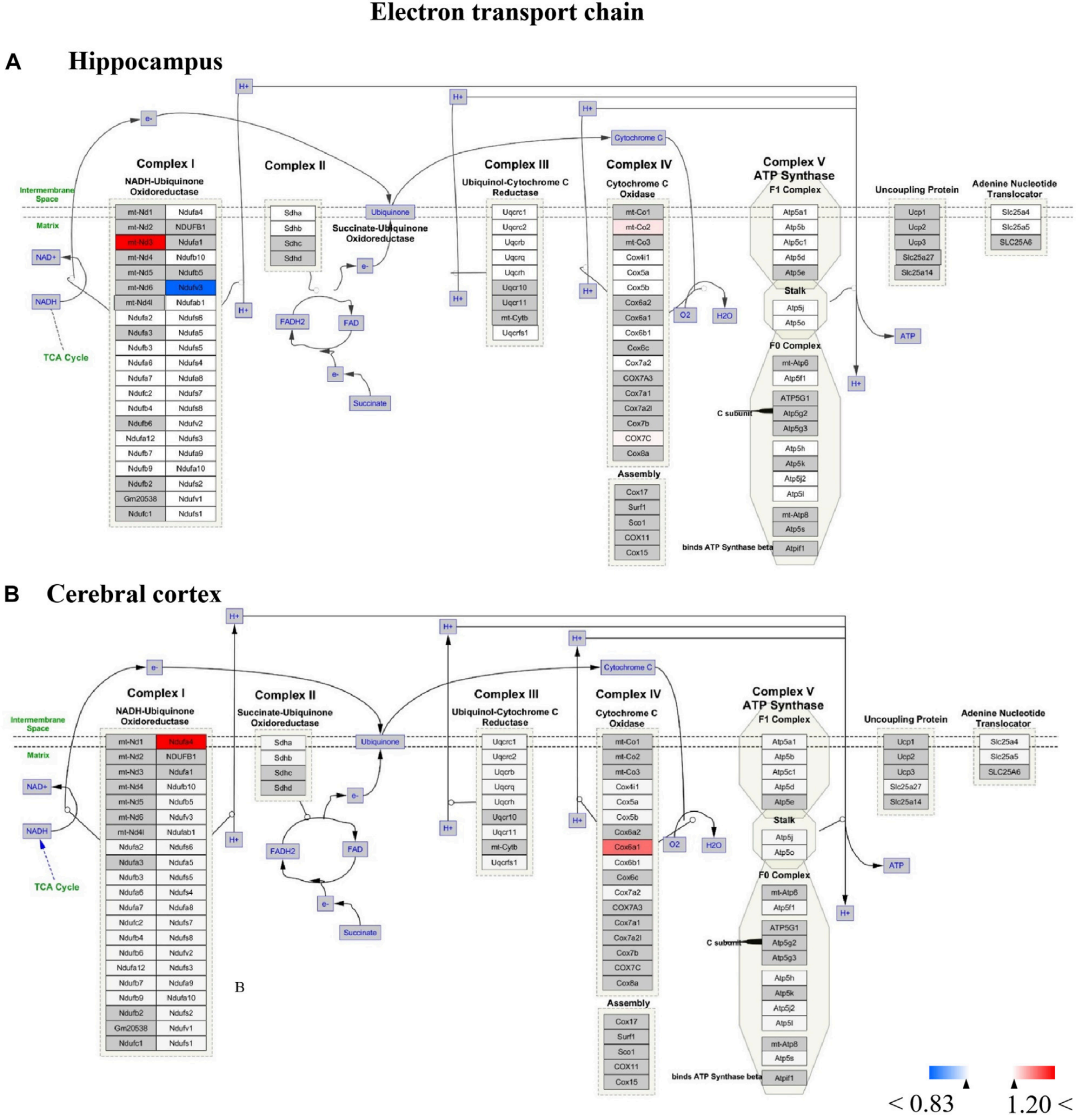
**(A,B)** All the identified proteins of hippocampus or cerebral cortex in the proteomics were imported into Cytoscape software to map electron transport chain based on the published database. The red boxes indicate up-regulated protein (ratio>1.2), blue boxes indicate down-regulated protein (ratio<0.83), white boxes indicate the indistinctively changed proteins and gray boxes stand for the unidentified proteins in this proteomic study.

Protein-protein interaction (PPI) networks and Wiki pathway were analyzed using Cytoscape 3.6.0 in order to evaluate the relationships among the altered proteins by TBN treatment in 3×Tg-AD mice (Figure 11; Figure 12). The result showed that more than half of the altered proteins were linked each other in the hippocampus and cerebral cortex, many of which were involved in AD pathology. As shown in Figure 11A, the most represented pathways in the hippocampus were oxidative phosphorylation, glutamate receptor activity, Ubiquitin pathway, MAPK signaling pathway, mTOR signaling pathway, cytoskeleton organization, ribosome and mitophagy. In the cerebral cortex (Figure 11B), the modulated pathways were more complex, including oxidative phosphorylation, ribosome, spliceosome, cytoskeleton organization, MAPK signaling pathway, AMPK-PI3K-Akt signaling pathway and metabolic pathway. These results revealed that TBN might modulate the multiple pathways in order to protect neuronal death.

**FIGURE 13:**
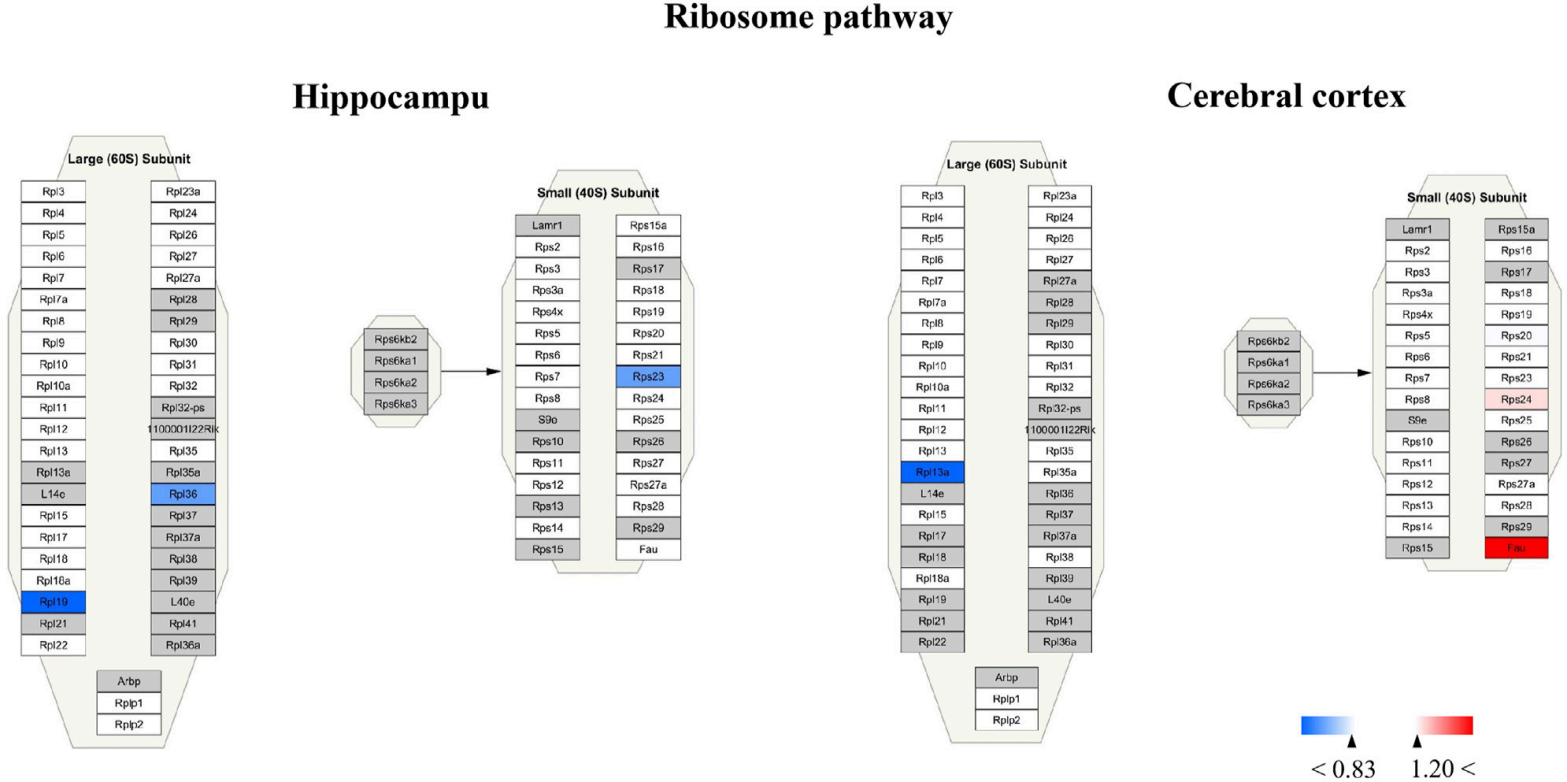
Visualization of altered protein by TBN treatment in the ribosome pathway between hippocampus and cerebral cortex. All the identified proteins of hippocampus or cerebral cortex in the proteomics were imported into Cytoscape software to map ribosome Wiki pathways based on the published database. The red boxes indicate upregulated protein (ratio ≥1.2), blue boxes indicate downregulated protein (ratio< 0.83), white boxes indicate the indistinctively changed proteins and gray boxes stand for the unidentified proteins in this proteomic study.

For Wiki pathway, it was observed that more than half of proteins of the electron transport chain was affected by TBN treatment (Figure 12). As Figure 12A shown that the expression of mt-Nd3 was significantly elevated and the expression of Ndufv3 of mitochondrial complex I was dramatically decreased in the hippocampus of TBN-treated 3×Tg-AD mice. However, in the cerebral cortex, TBN could significantly increase the levels of Ndufa4 protein in the complex I and Cox6a1 protein in the complex IV, and do not lower any protein expression (Figure 12 B). In addition, the results from cytoplasmic ribosomal proteins pathway analysis indicated most of ribosomal protein subunits were influenced by TBN treatment in the hippocampus or cerebral cortex of 3×Tg-AD mice (Figure 13). In the hippocampus, protein Rpl 19, protein Rpl 36 and protein Rps 23 was significantly downregulated. Whereas, in the cerebral cortex, only protein Rpl 13a was dramatically downregulated, and protein Fau was significantly upregulated. The results indicated that the modulation of TBN on electron transport chain and ribosome pathway was dependent on different brain region associating with learning and memory behavior.

### 3.8 Identification of differentially expressed proteins in the hippocampus of 3×Tg-AD mice by Western blot analysis

**FIGURE 14:**
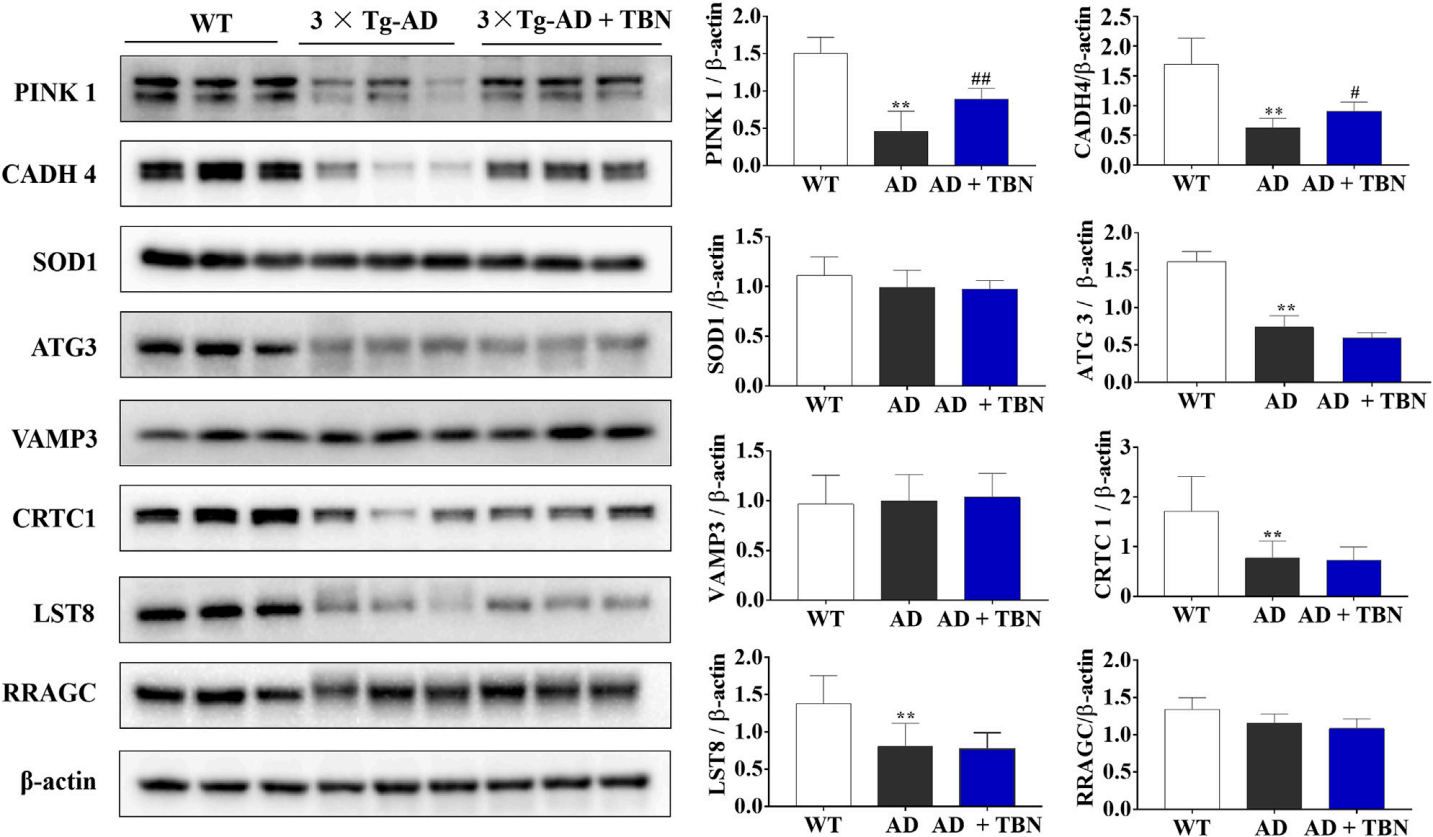
Validation of altered proteins identified by Western blot. Samples of hippocampal tissues (same samples as in proteomic study) were evaluated by Western blot. Representative blotting of PINK1, CADH4, SOD1, ATG3, VAMP3, CRTC1, LST8 and RRAGC in the hippocampal tissues of AD mice or WT mice were showed. Data were presented as mean ± SEM, n ≥ 6, **p < 0.01 vs. WT group, ^*#*^ *p* < 0.05 or ^*##*^ *p* < 0.01 vs. AD group, one-way ANOVA.

To further verify results from proteomic analysis, the samples of hippocampal tissues of 3×Tg-AD mice or WT mice was used to performed Western blot assays (Figure 14). Base on proteomic analysis, PINK1 (ratio: 1.62), CADH 4 (ratio: 1.53), VAMP3 (ratio: 1.51), SOD1 (ratio: 1.45), ATG3 (ratio: 1.26), Crtc1 (ratio: 1.2), LST8 (ratio: 0.75) and RRAGC (ratio: 0.64) were selected for further study (Table 1). As shown in Figure 14, the levels of PINK1 and CADH4 were significantly increased in TBN-treated group when compared with AD control group, which was consistent with the results of proteomic analysis that TBN significantly upregulated the expression of PINK1 and CADH4 (Table 1). However, the Western blot results of protein SOD1, ATG 3, VAMP 3, CRTC1, LST8 and RRAGC indicated that TBN had no effects on regulating their expression levels.

**TABLE 1:** The information of validated proteins in the hippocampus.

Accession	Protein name	Description	Ratio			
			WT	AD/WT	TBN/WT	TBN/AD
Q99MQ3	PINK1	Serine/threonine-protein kinase PINK1 mitochondrial,GN = Pink1	1	0.73	1.18	1.62
P39038	CADH4	Cadherin-4,GN = Cdh4	1	0.58	0.89	1.53
P08228	SOD1	Superoxide dismutase [Cu-Zn],GN = Sod1	1	0.99	1.44	1.45
Q9CPX6	ATG3	Ubiquitin-like-conjugating enzyme ATG3,GN = Atg3	1	0.78	0.98	1.26
P63024	VAMP3	Vesicle-associated membrane protein 3,GN = Vamp3	1	0.71	1.07	1.51
Q68ED7	CRTC1	CREB-regulated transcription coactivator 1,GN = Crtc1	1	0.85	1.02	1.20
Q9DCJ1	LST8	Target of rapamycin complex subunit LST8,GN = Mlst8	1	1.16	0.87	0.75
Q99K70	RRAGC	Ras-related GTP-binding protein C,GN = Rragc	1	1.12	0.72	0.64

Note: GN, means abbreviation of gene name.

## 4 Discussion

Safe and effective pharmacological treatments capable of stopping or at least delaying the course of AD are still under extensive research. TBN is a promising candidate that has recently been approved for the Phase II clinical trial for ischemic stroke (Zhang et al., 2016). In this study, we aimed at further evaluating the neuroprotective effects of TBN in well-established AD related models. We demonstrated that TBN significantly reduced Aβ production and deposition, attenuated tau hyperphosphorylation, and prevented neuronal dendritic spine loss, all of which probably contributed to the significant improvement of learning and memory in 3×Tg-AD mouse model. *In vitro* data from N2a/APPswe cells suggested the overproduction of Aβ in N2a/APPswe cells were mainly caused by elevated APP production, and possibly by downregulation of ADAM10. More interestingly, TBN not only significantly reversed the altered levels of APP and ADAM10 in N2a/APPswe, but also suppressed the expressions of BACE1 and PS1 (Figure 1C), reduced the production of sAPPβ and CTFβ (amyloidogenic pathway) and increased the production of CTFα (non-amyloidogenic pathway) (Figures 1E, F). However, TBN had negligible effects on the expressions of IDE and NEP (Figure 1G, H). These data indicated that TBN reduced Aβ reduction mainly *via* reducing total APP levels and modulating APP processing pathways rather than degradation. The data from N2a/APPswe cells also showed that TBN markedly reduced tau hyperphosphrylation. Taken together, these data indicate that TBN significantly reduced total APP and Aβ levels, modulated APP processing through attenuating amyloidogenic (PS1 and BACE1) and enhancing non-amyloidogenic (ADAM10) expression, as well as reducing tau hyperphosphrylation.

In early-onset AD, overproduction of Aβ is caused by mutations in the amyloid precursor protein (APP) gene at the *ß*-secretase cleavage site driving dysregulation of the amyloidogenic pathway. Meanwhile, impairment of Aβ degradation further aggravates Aβ clearance (Miners et al., 2011). The proteolytic degradation of Aβ by Aβ-degrading enzymes (ADE) is a major route of Aβ clearance (Nalivaeva et al., 2008). Among the ADE, neprilysin (NEP) and insulin-degrading enzyme (IDE) are considered two most important enzymes for regulation of cerebral Aβ levels. NEP degrades soluble Aβ monomers and oligomers while IDE only degrades soluble Aβ monomers (Jha et al., 2015; Ries & Sastre, 2016). It has been demonstrated that the level and activity of NEP was significantly reduced in the cortex and hippocampus of AD brain, and irregular function of NEP causes a two-fold increase in the endogenous toxic Aβ40-42 levels within the brain, which lead to impaired synaptic plasticity and cognitive abnormalities that ultimately increase the risk of AD (Iwata et al.,2005; Jha et al., 2015). Similarly, the level and activity of IDE was reduced in the hippocampus of individuals with mild cognitive impairment (MCI), which was correlated with the conversion from MCI to dementia (Zhao et al., 2007). Previous studies demonstrated overexpression of IDE or NEP in neurons in APP transgenic mice *in vivo* rescued premature lethality by upregulating Aβ degradation which significantly reduced brain Aβ levels and prevented plaque formation (Leissring et al., 2003; Turner etl, 2004). Our study results show that TBN could decrease the levels of APP, BACE1 and PS1, and increase the levels of NEP, indicated that TBN prevents the amyloid plaque formation and enhances amyloid degradation, which account for the reduction levels of Aβ production and deposition in 3×Tg-AD mouse model.

Our results from proteomics profiling indicated that TBN had neuroprotective effects associated with multiple pathways, including the modulation of mitophagy, MAPK signaling pathway and mTOR signaling pathway. Proteomics analysis also indicated that TBN notably regulated proteins relevant to AD. Notably, TBN promoted the expression of PINK1, a downstream effector of parkin in a pathway that affects mitochondrial homeostasis. Consistent with our previous studies, we observed a significant reduction of the expression of PINK1 in the hippocampal tissues of 3×Tg-AD mice, which might be a potential therapeutic target for AD (Zhou et al., 2019a). We further confirmed that TBN significantly increased PINK1 protein expression in the hippocampal tissues of 3×Tg-AD mice by using Western blot (Figure 14). Parkin/PINK1 has been shown to mediate mitophagy, a selective form of autophagy for removing damaged mitochondria. Mitochondrial quality control mechanisms such as mitophagy play indispensable roles in the preservation of neuronal health, and studies have shown that various proteins related to mitochondrial quality control (e.g., mitophagy, mitochondria biogenesis and mitochondrial dynamics) are affected in AD (Rodolfo, Campello, & Cecconi, 2018). In this regard, accumulation of dysfunctional/damaged mitochondria has been reported as an initial symptom of AD, which further contributing to disease progression such as intracellular calcium imbalance, oxidative stress, Aβ and tau pathologies (Ashleigh, Swerdlow, & Beal, 2022). In particular, PINK1 has been associated with the onset and progression of many neurodegenerative diseases including AD, and PINK1 downregulation leads to mitochondrial dysfunction, increase oxidative stress, and neuronal dysfunction (Tufi et al., 2014; Rodolfo et al., 2018). Furthermore, Mitochondria dysfunction is also linked to misfolded protein aggregation such as Aβ and hyperphosphorylated tau (Kerr et al., 2017; Rodolfo et al., 2018). A previous study using AD-related mouse model showed that PINK1 was associated with synaptic plasticity and its over-expression significantly improved cognitive functions, while under-expression of PINK1 enhanced Aβ accumulation and exacerbated mitochondrial and synaptic damages (F. Du et al., 2017). Consistent with our previous observation, these data showed significant hippocampal PINK1 upregulation in 3×Tg-AD mice treated with TBN.

Many hypotheses have been put forward for the pathogenesis of AD, and it is now accepted that AD is a multi-modal neurodegenerative disorder with the earliest cellular phase when pernicious biochemical changes occur in the brain inducing gradual and irreversible harmful effects. Moreover, it is suggested that Aβ and tau pathology happen in parallel with these cellular changes and do not act in isolation. Targeting all these disease-related factors might provide a more effective way of AD intervention. Here, we demonstrated that TBN is a multifunctional agent capable of ameliorating Aβ and tau pathologies, promoting mitochondrial homeostasis, protecting neuronal damages, and improving cognitive abilities. In addition, TBN showed significant therapeutic efficacy treating ischemic stroke with proven safety in preclinical studies. Therefore, we believe that TBN is a promising anti-AD agent with potential disease-modifying therapeutic values.

## Data Availability

The datasets presented in this study can be found in online repositories. The names of the repository/repositories and accession number(s) can be found in the article/Supplementary Material.
