# Supplementary material for: Evaluation of therapeutic effects of tetramethylpyrazine nitrone in Alzheimer’s disease mouse model and proteomics analysis
Source: Front Pharmacol. 2023 Mar 6;14:1082602. doi: 10.3389/fphar.2023.1082602 (PMC10025301; doi:10.3389/fphar.2023.1082602)
Supplement: Supplementary file 1 [file DataSheet1.docx]

# Supplementary Data

Table A-1. The 32 proteins that were changed significantly by TBN treatment in the hippocampus of 3 × Tg-AD mice. These proteins have met the criteria, the ratio of TBN / AD in expression levels of at least 1.5-fold (up-regulation) or at least＜0.67-fold (down-regulation) as defined in the experimental procedures.

| Accession | Protein name | Description | Ratio | | | #Unique |
| --- | --- | --- | --- | --- | --- | --- |
|  |  |  | AD/WT | TBN/WT | TBN/AD |  |
| O70311 | NMT2 | Glycylpeptide N-tetradecanoyltransferase 2, GN=Nmt2 | 0.88 | 2.1 | 2.39 | 2 |
| P03899 | NU3M | NADH-ubiquinone oxidoreductase chain 3, GN=Mtnd3 | 0.86 | 1.85 | 2.15 | 2 |
| Q60974 | NCOR1 | Nuclear receptor corepressor 1, GN=Ncor1 | 0.63 | 1.25 | 1.98 | 1 |
| Q80WM5 | HPLN3 | Hyaluronan and proteoglycan link protein 3, GN=Hapln3 | 1.01 | 1.89 | 1.87 | 1 |
| Q8BGX2 | TIM29 | Mitochondrial import inner membrane translocase subunit Tim29, GN=Hapln3 | 0.53 | 0.92 | 1.74 | 1 |
| Q07076 | ANXA7 | Annexin A7, GN=Anxa7 | 1.11 | 1.86 | 1.68 | 7 |
| Q810U3 | NFASC | Neurofascin, GN=Nfasc | 1.01 | 1.69 | 1.67 | 1 |
| Q8BG37 | Q8BG37 | MCG48959, GN=Peroxiredoxin 6, GN= related sequence 1, GN=Prdx6b | 0.74 | 1.22 | 1.65 | 1 |
| Q99MQ3 | PINK1 | Serine/threonine-protein kinase PINK1 mitochondrial, GN=Pink1 | 0.73 | 1.18 | 1.62 | 1 |
| O88491 | NSD1 | Histone-lysine N-methyltransferase H3 lysine-36 and H4 lysine-20 specific, GN=Nsd1 | 0.76 | 1.17 | 1.54 | 1 |
| P39038 | CADH4 | Cadherin-4, GN=Cdh4 | 0.58 | 0.89 | 1.53 | 1 |
| P63024 | VAMP3 | Vesicle-associated membrane protein 3, GN=Vamp3 | 0.71 | 1.07 | 1.51 | 1 |
| O08967 | CYH3 | Cytohesin-3, GN=Cyth3 | 0.96 | 0.64 | 0.67 | 1 |
| G5E866 | G5E866 | Splicing factor 3B subunit 1, GN=Sf3b1 | 1.38 | 0.92 | 0.67 | 1 |
| P62077 | TIM8B | Mitochondrial import inner membrane translocase subunit Tim8 B, GN=Timm8b | 1.16 | 0.77 | 0.66 | 1 |
| P26350 | PTMA | Prothymosin alpha, GN=Ptma | 1.07 | 0.71 | 0.66 | 2 |
| Q6WQJ1 | DGLA | Sn1-specific diacylglycerol lipase alpha, GN=Dagla | 1.01 | 0.67 | 0.66 | 1 |
| Q9EQP2 | EHD4 | EH domain-containing protein 4, GN=Ehd4 | 1.01 | 0.67 | 0.66 | 1 |
| Q9JKL4 | NDUF3 | NADH dehydrogenase [ubiquinone] 1 alpha subcomplex assembly factor 3, GN=Ndufaf3 | 1.72 | 1.14 | 0.66 | 2 |
| Q80UK8 | INT2 | Integrator complex subunit 2, GN=Ints2 | 1.32 | 0.87 | 0.66 | 1 |
| Q99MR0 | ACL6B | Actin-like protein 6B, GN=Actl6b | 1.33 | 0.86 | 0.65 | 1 |
| Q99K70 | RRAGC | Ras-related GTP-binding protein C, GN=Rragc | 1.12 | 0.72 | 0.64 | 1 |
| Q8R395 | COMD5 | COMM domain-containing protein 5, GN=Commd5 | 1.07 | 0.68 | 0.64 | 1 |
| Q99P58 | RB27B | Ras-related protein Rab-27B, GN=Rab27b | 1.69 | 1.07 | 0.63 | 1 |
| Q9ERI2 | RB27A | Ras-related protein Rab-27A, GN=Rab27a | 1.69 | 1.07 | 0.63 | 1 |
| E9PYG6 | E9PYG6 | RAS p21 protein activator 1,GN=Rasa1 | 1.25 | 0.79 | 0.63 | 1 |
| Q3U285 | LCORL | Ligand-dependent nuclear receptor corepressor-like protein, GN=Lcorl | 1.61 | 0.97 | 0.60 | 1 |
| P62806 | H4 | Histone H4, GN=Hist1h4a | 0.86 | 0.5 | 0.58 | 8 |
| Q8BUK6 | HOOK3 | Protein Hook homolog 3, GN=Hook3 | 0.78 | 0.45 | 0.58 | 1 |
| F6QFD1 | F6QFD1 | Phosphodiesterase (Fragment), GN=Pde4d | 1.47 | 0.82 | 0.56 | 2 |
| E9Q3H7 | E9Q3H7 | Dynein axonemal heavy chain 6, GN=Dnah6 | 2.48 | 1.27 | 0.51 | 1 |
| P19070 | CR2 | Complement receptor type 2, GN=Cr2 | 1.63 | 0.83 | 0.51 | 1 |

Table A-2. The 74 proteins that were changed significantly by TBN treatment in the cerebral cortex of 3 × Tg-AD mice. These proteins have met the criteria, the ratio of TBN / AD in expression levels of at least 1.5-fold (up-regulation) or at least ＜0.67-fold (down-regulation) as defined in the experimental procedures.

| Accession | Protein name | Description | Ratio | | |  | |
| --- | --- | --- | --- | --- | --- | --- | --- |
|  |  |  | AD/WT | TBN/WT | TBN/AD | | #Unique |
| Q7M6Y3 | PICAL | Phosphatidylinositol-binding clathrin assembly protein, GN=Picalm | 0.47 | 0.86 | 1.83 | | 1 |
| P62073 | TIM10 | Mitochondrial import inner membrane translocase subunit Tim10, GN=Timm10 | 0.94 | 1.61 | 1.71 | | 2 |
| Q9Z2D3 | DFNA5 | Non-syndromic hearing impairment protein 5 homolog, GN=Dfna5 | 0.84 | 1.31 | 1.56 | | 1 |
| E9PVS5 | E9PVS5 | MICOS complex subunit MIC60 (Fragment), GN=Immt | 0.98 | 1.5 | 1.53 | | 1 |
| Q64669 | NQO1 | NAD(P)H dehydrogenase [quinone] 1, GN=Nqo1 | 0.79 | 1.19 | 1.51 | | 1 |
| Q9CQ02 | COMD4 | COMM domain-containing protein 4, GN=Commd4 | 0.76 | 1.14 | 1.50 | | 1 |
| Q9JHL1 | NHRF2 | Na (+)/H (+) exchange regulatory cofactor NHE-RF2, GN=Slc9a3r2 | 1.38 | 0.92 | 0.67 | | 2 |
| Q68FD9 | K1549 | UPF0606 protein KIAA1549, GN=Kiaa1549 | 1.48 | 0.98 | 0.66 | | 3 |
| O70274 | TP4A2 | Protein tyrosine phosphatase type IVA 2, GN=Ptp4a2 | 1.12 | 0.74 | 0.66 | | 1 |
| P49070 | CAMLG | Calcium signal-modulating cyclophilin ligand, GN=Camlg | 1.06 | 0.7 | 0.66 | | 1 |
| Q60902 | EP15R | Epidermal growth factor receptor substrate 15-like 1, GN=Eps15l1 | 1.53 | 1.01 | 0.66 | | 11 |
| A0A0R4J034 | A0A0R4J034 | MCG129810 isoform CRA_c, GN=Pdxdc1 | 1 | 0.66 | 0.66 | | 2 |
| Q9CXT8 | MPPB | Mitochondrial-processing peptidase subunit beta, GN=Pmpcb | 1.28 | 0.84 | 0.66 | | 1 |
| P38585 | TTL | Tubulin--tyrosine ligase, GN=Ttl | 1.27 | 0.83 | 0.65 | | 1 |
| O54724 | CAVN1 | Caveolae-associated protein 1, GN=Cavin1 | 1.52 | 0.99 | 0.65 | | 3 |
| E9QAC6 | E9QAC6 | Galectin, GN=Pick1 | 1.97 | 1.28 | 0.65 | | 1 |
| E9PYX6 | E9PYX6 | Sorbin and SH3 domain-containing protein 1, GN=Sorbs1 | 1.54 | 1 | 0.65 | | 1 |
| E9QAS7 | E9QAS7 | Inositol polyphosphate-5-phosphatase A, GN=Inpp5a | 1.65 | 1.07 | 0.65 | | 2 |
| Q9D8S9 | BOLA1 | BolA-like protein 1, GN=Bola1 | 1.53 | 0.99 | 0.65 | | 2 |
| Q8K327 | CHAP1 | Chromosome alignment-maintaining phosphoprotein 1, GN=Champ1 | 1.47 | 0.95 | 0.65 | | 2 |
| Q6PAK3 | ANM8 | Protein arginine N-methyltransferase 8, GN=Prmt8 | 1.51 | 0.97 | 0.64 | | 1 |
| P19253 | RL13A | 60S ribosomal protein L13a , GN=Rpl13a | 1.5 | 0.96 | 0.64 | | 3 |
| Q8BHZ0 | FA49A | Protein FAM49A, GN=Fam49a | 0.91 | 0.58 | 0.64 | | 1 |
| Q8BGJ0 | ZDH15 | Palmitoyltransferase ZDHHC15, GN=Zdhhc15 | 1.93 | 1.23 | 0.64 | | 1 |
| Q9Z315 | SNUT1 | U4/U6. U5 tri-snRNP-associated protein 1, GN=Sart1 | 0.93 | 0.59 | 0.63 | | 1 |
| Q9CYI4 | LUC7L | Putative RNA-binding protein Luc7-like 1, GN=Luc7l | 1.22 | 0.77 | 0.63 | | 2 |
| Q9QZB1 | RGS20 | Regulator of G-protein signaling 20, GN=Rgs20 | 1.49 | 0.94 | 0.63 | | 3 |
| Q9WTU6 | MK09 | Mitogen-activated protein kinase 9, GN=Mapk9 | 1.57 | 0.99 | 0.63 | | 2 |
| Q64288 | OMP | Olfactory marker protein, GN=Omp | 1.24 | 0.78 | 0.63 | | 4 |
| Q8BVG4 | DPP9 | Dipeptidyl peptidase 9, GN=Dpp9 | 1.21 | 0.76 | 0.63 | | 1 |
| Q62167 | DDX3X | ATP-dependent RNA helicase DDX3X, GN=Ddx3x | 1.93 | 1.19 | 0.62 | | 1 |
| A0JP43 | EFCB5 | EF-hand calcium-binding domain-containing protein 5, GN=Efcab5 | 1.55 | 0.95 | 0.61 | | 1 |
| O70340 | NPTX2 | Neuronal pentraxin-2, GN=Nptx2 | 1.42 | 0.87 | 0.61 | | 1 |
| P0C0A3 | CHMP6 | Charged multivesicular body protein 6, GN=Chmp6 | 1.39 | 0.85 | 0.61 | | 1 |
| Q14BI7 | TDRD9 | Putative ATP-dependent RNA helicase TDRD9, GN=Tdrd9 | 1.93 | 1.18 | 0.61 | | 1 |
| Q8VHJ5 | MARK1 | Serine/threonine-protein kinase MARK1, GN=Mark1 | 1.52 | 0.92 | 0.61 | | 2 |
| P32883 | RASK | GTPase KRas, GN=Kras | 1.45 | 0.87 | 0.60 | | 1 |
| Q3TCJ1 | ABRX2 | BRISC complex subunit Abraxas 2, GN=Abraxas2 | 1.42 | 0.85 | 0.60 | | 1 |
| P54754 | EPHB3 | Ephrin type-B receptor 3, GN=Ephb3 | 1.22 | 0.73 | 0.60 | | 1 |
| Q9QZE5 | COPG1 | Coatomer subunit gamma-1, GN=Copg1 | 1.66 | 0.98 | 0.59 | | 4 |
| Q9CS84 | NRX1A | Neurexin-1, GN=Nrxn1 | 1.7 | 1 | 0.59 | | 11 |
| Q6ZQ18 | EFR3B | Protein EFR3 homolog B, GN=Efr3b | 1.6 | 0.94 | 0.59 | | 6 |
| O88587 | COMT | Catechol O-methyltransferase, GN=Comt | 2.24 | 1.3 | 0.58 | | 1 |
| O88983 | STX8 | Syntaxin-8, GN=Stx8 | 1.3 | 0.75 | 0.58 | | 1 |
| A0A1L1SQG7 | A0A1L1SQG7 | Ryanodine receptor 1, GN=Ryr1 | 1.25 | 0.72 | 0.58 | | 2 |
| O08808 | DIAP1 | Protein diaphanous homolog 1, GN=Diaph1 | 1.58 | 0.91 | 0.58 | | 1 |
| Q6P542 | ABCF1 | ATP-binding cassette sub-family F member 1, GN=Abcf1 | 1.55 | 0.89 | 0.57 | | 2 |
| P43274 | H14 | Histone H1.4, GN=Hist1h1e | 1.29 | 0.74 | 0.57 | | 1 |
| G3XA57 | RFIP2 | Rab11 family-interacting protein 2, GN=Rab11fip2 | 1.59 | 0.9 | 0.57 | | 2 |
| Q9D3R6 | KATL2 | Katanin p60 ATPase-containing subunit A-like 2, GN=Katnal2 | 1.31 | 0.74 | 0.56 | | 1 |
| Q91ZR1 | RAB4B | Ras-related protein Rab-4B, GN=Rab4b | 1.63 | 0.92 | 0.56 | | 2 |
| A0A0A6YY91 | A0A0A6YY91 | Neural cell adhesion molecule 1 (Fragment), GN=Ncam1 | 2.1 | 1.17 | 0.56 | | 2 |
| O35166 | GOSR2 | Golgi SNAP receptor complex member 2, GN=Gosr2 | 1.01 | 0.56 | 0.55 | | 1 |
| E9Q0A7 | E9Q0A7 | Oxidation resistance protein 1, GN=Oxr1 | 1.76 | 0.95 | 0.54 | | 1 |
| Q9D1M0 | SEC13 | Protein SEC13 homolog, GN=Sec13 | 1.86 | 1 | 0.54 | | 1 |
| Q810A7 | DDX42 | ATP-dependent RNA helicase DDX42, GN=Ddx42 | 1.6 | 0.86 | 0.54 | | 1 |
| Q9WTS5 | TEN2 | Teneurin-2, GN=Tenm2 | 1.66 | 0.86 | 0.52 | | 1 |
| Q9EPE9 | AT131 | Manganese-transporting ATPase 13A1, GN=Atp13a1 | 2.02 | 1 | 0.50 | | 1 |
| Q68FH0 | PKP4 | Plakophilin-4, GN=Pkp4 | 2.33 | 1.15 | 0.49 | | 4 |
| E9PUD2 | E9PUD2 | Dynamin-1-like protein, GN=Dnm1l | 1.54 | 0.76 | 0.49 | | 1 |
| Q9D061 | ACBD6 | Acyl-CoA-binding domain-containing protein 6, GN=Acbd6 | 1.73 | 0.84 | 0.49 | | 4 |
| Q6PEV3 | WIPF2 | WAS/WASL-interacting protein family member 2, GN=Wipf2 | 2 | 0.97 | 0.49 | | 1 |
| Q61161 | M4K2 | Mitogen-activated protein kinase kinase kinase kinase 2, GN=Map4k2 | 1.89 | 0.88 | 0.47 | | 3 |
| O35343 | IMA3 | Importin subunit alpha-3, GN=Kpna4 | 2.24 | 1.02 | 0.46 | | 1 |
| Q9JIY5 | HTRA2 | Serine protease HTRA2 mitochondrial, GN=Htra2 | 2.81 | 1.18 | 0.42 | | 1 |
| A2AG36 | A2AG36 | GPI ethanolamine phosphate transferase 3, GN=Pigo | 2.03 | 0.85 | 0.42 | | 1 |
| Q8R1S0 | COQ6 | Ubiquinone biosynthesis monooxygenase COQ6 mitochondrial, GN=Coq6 | 2.68 | 1.01 | 0.38 | | 3 |
| F2Z483 | F2Z483 | T-complex protein 1 subunit alpha, GN=Tcp1 | 2.96 | 1.08 | 0.36 | | 1 |
| A0A140LJB0 | A0A140LJB0 | Espin, GN=Espn | 2.59 | 0.91 | 0.35 | | 1 |
| E9PUU8 | E9PUU8 | Fibrocystin-L, GN=Pkhd1l1 | 3.42 | 1.03 | 0.30 | | 1 |
| Q7SIG6 | ASAP2 | Arf-GAP with SH3 domain ANK repeat and PH domain-containing protein 2, GN=Asap2 | 3.38 | 1 | 0.30 | | 2 |
| Q8VHR5 | P66B | Transcriptional repressor p66-beta, GN=Gatad2b | 3.57 | 0.83 | 0.23 | | 1 |
| A6ZI46 | A6ZI46 | Fructose-bisphosphate aldolase, GN=Aldoart1 | 4.36 | 0.85 | 0.19 | | 1 |
| Q9WVL3 | S12A7 | Solute carrier family 12 member 7, GN=Slc12a7 | 27.85 | 0.8 | 0.03 | | 1 |

**Table A-3. The 32 proteins that were changed significantly by TBN treatment in both the hippocampus and cortex of 3 × Tg-AD mice.** These proteins have met the criteria, the ratio of TBN / AD in expression levels of at least 1.2-fold (up-regulation) or at least＜0.83-fold (down-regulation) as defined in the experimental procedures.

| Accession | Protein name | Description | Hippocampus | | | | Cerebral cortex | | | |
| --- | --- | --- | --- | --- | --- | --- | --- | --- | --- | --- |
|  |  |  | AD/WT | TBN/WT | TBN/AD | unique | AD/WT | TBN/WT | TBN/AD | unique |
| Q9EPL8 | IPO7 | Importin-7, GN=Ipo7 | 0.86 | 1.25 | 1.45 | 2 | 1.16 | 0.94 | 0.81 | 4 |
| E0CY16 | E0CY16 | Cell adhesion molecule 1, GN=Cadm1 | 1.06 | 1.51 | 1.42 | 1 | 1.26 | 0.89 | 0.71 | 1 |
| Q8BYP3 | RHOF | Rho-related GTP-binding protein RhoF, GN=Rhof | 0.87 | 1.23 | 1.41 | 1 | 1.26 | 0.96 | 0.76 | 1 |
| P07356 | ANXA2 | Annexin A2, GN=Anxa2 | 0.81 | 1.11 | 1.37 | 1 | 1.31 | 1.08 | 0.82 | 3 |
| Q8BH69 | SPS1 | Selenide water dikinase 1, GN=Sephs1 | 0.71 | 0.96 | 1.35 | 1 | 1.17 | 0.83 | 0.71 | 1 |
| Q9D0F6 | RFC5 | Replication factor C subunit 5, GN=Rfc5 | 1.06 | 1.33 | 1.25 | 1 | 1.21 | 0.87 | 0.72 | 1 |
| Q9D061 | ACBD6 | Acyl-CoA-binding domain-containing protein 6, GN=Acbd6 | 0.88 | 1.08 | 1.23 | 2 | 1.73 | 0.84 | 0.49 | 4 |
| Q62376 | RU17 | U1 small nuclear ribonucleoprotein 70 kDa, GN=Snrnp70 | 0.89 | 1.07 | 1.20 | 2 | 1.22 | 1.00 | 0.82 | 4 |
| Q07076 | ANXA7 | Annexin A7, GN=Anxa7 | 1.11 | 1.86 | 1.68 | 7 | 0.96 | 1.30 | 1.35 | 10 |
| P08228 | SODC | Superoxide dismutase [Cu-Zn], GN=Sod1 | 0.99 | 1.44 | 1.45 | 6 | 1.09 | 1.47 | 1.35 | 6 |
| P00405 | COX2 | Cytochrome c oxidase subunit 2, GN=Mtco2 | 1.00 | 1.29 | 1.29 | 5 | 1.06 | 1.32 | 1.25 | 6 |
| P17665 | COX7C | Cytochrome c oxidase subunit 7C mitochondrial, GN=Cox7c | 0.93 | 1.15 | 1.24 | 1 | 0.68 | 0.95 | 1.40 | 2 |
| A0A140LHQ8 | A0A140LHQ8 | Phosphatidylinositol-binding clathrin assembly protein (Fragment), GN=Picalm | 0.90 | 1.09 | 1.21 | 1 | 0.53 | 0.68 | 1.28 | 1 |
| Q91ZP9 | NECA2 | N-terminal EF-hand calcium-binding protein 2, GN=Necab2 | 1.14 | 0.94 | 0.82 | 5 | 1.21 | 0.95 | 0.79 | 4 |
| Q61941 | NNTM | NAD(P) transhydrogenase mitochondrial, GN=Nnt | 1.66 | 1.36 | 0.82 | 7 | 1.55 | 1.24 | 0.80 | 9 |
| Q9R1Z7 | PTPS | 6-pyruvoyl tetrahydrobiopterin synthase, GN=Pts | 1.31 | 1.07 | 0.82 | 2 | 1.27 | 1.02 | 0.80 | 1 |
| Q99JB2 | STML2 | Stomatin-like protein 2 mitochondrial, GN=Stoml2 | 1.05 | 0.85 | 0.81 | 4 | 1.10 | 0.87 | 0.79 | 4 |
| P23819 | GRIA2 | Glutamate receptor 2, GN=Gria2 | 1.24 | 1.00 | 0.81 | 1 | 1.20 | 0.91 | 0.76 | 1 |
| Q60996 | 2A5G | Serine/threonine-protein phosphatase 2A 56 kDa regulatory subunit gamma isoform, GN=Ppp2r5c | 1.29 | 1.04 | 0.81 | 1 | 1.12 | 0.79 | 0.71 | 2 |
| P60843 | IF4A1 | Eukaryotic initiation factor 4A-I, GN=Eif4a1 | 1.17 | 0.94 | 0.80 | 4 | 1.17 | 0.96 | 0.82 | 2 |
| Q8K4Z0 | LGI2 | Leucine-rich repeat LGI family member 2, GN=Lgi2 | 0.96 | 0.77 | 0.80 | 1 | 1.40 | 0.97 | 0.69 | 3 |
| P97855 | G3BP1 | Ras GTPase-activating protein-binding protein 1, GN=G3bp1 | 1.23 | 0.98 | 0.80 | 3 | 0.94 | 0.72 | 0.77 | 3 |
| A8DUK4 | A8DUK4 | Beta-globin, GN=Hbbt1 | 0.58 | 0.46 | 0.79 | 3 | 0.72 | 0.43 | 0.60 | 5 |
| Q924N4 | S12A6 | Solute carrier family 12 member 6, GN=Slc12a6 | 1.01 | 0.80 | 0.79 | 1 | 1.70 | 1.31 | 0.77 | 1 |
| P84244 | H33 | Histone H3.3, GN=H3f3a | 0.76 | 0.60 | 0.79 | 1 | 0.87 | 0.68 | 0.78 | 1 |
| Q3V0K9 | PLSI | Plastin-1, GN=Pls1 | 1.13 | 0.88 | 0.78 | 1 | 0.91 | 0.72 | 0.79 | 2 |
| Q8BTI8 | SRRM2 | Serine/arginine repetitive matrix protein 2, GN=Srrm2 | 0.93 | 0.70 | 0.75 | 1 | 1.61 | 1.19 | 0.74 | 1 |
| A2AQ07 | TBB1 | Tubulin beta-1 chain, GN=Tubb1 | 1.10 | 0.82 | 0.75 | 2 | 1.05 | 0.77 | 0.73 | 1 |
| Q9D1F4 | AKTS1 | Proline-rich AKT1 substrate 1, GN=Akt1s1 | 1.06 | 0.73 | 0.69 | 1 | 0.86 | 0.64 | 0.74 | 1 |
| G5E866 | G5E866 | Splicing factor 3B subunit 1, GN=Sf3b1 | 1.38 | 0.92 | 0.67 | 1 | 1.21 | 0.91 | 0.75 | 1 |
| A2ARZ3 | FSIP2 | Fibrous sheath-interacting protein 2, GN=Fsip2 | 1.14 | 0.81 | 0.71 | 3 | 0.71 | 0.90 | 1.27 | 2 |
| Q6WQJ1 | DGLA | Sn1-specific diacylglycerol lipase alpha, GN=Dagla | 1.01 | 0.67 | 0.66 | 1 | 0.85 | 1.05 | 1.24 | 1 |
